# Identification of Gut Microbial Lysine and Histidine Degradation and CYP-Dependent Metabolites as Biomarkers of Fatty Liver Disease

**DOI:** 10.1128/mbio.02663-22

**Published:** 2023-01-30

**Authors:** Anastasiia Driuchina, Jukka Hintikka, Marko Lehtonen, Pekka Keski-Rahkonen, Thomas O’Connell, Risto Juvonen, Juho Kuula, Antti Hakkarainen, Jari A. Laukkanen, Elina Mäkinen, Sanna Lensu, Kirsi H. Pietiläinen, Satu Pekkala

**Affiliations:** a Faculty of Sport and Health Sciences, University of Jyväskylä, Jyväskylä, Finland; b Faculty of Health Sciences, School of Pharmacy, University of Eastern Finland, Kuopio, Finland; c Nutrition and Metabolism Branch, International Agency for Cancer on Research, Lyon, France; d Indiana University School of Medicine, Indianapolis, Indiana, USA; e HUS Medical Imaging Center, Department of Radiology, University of Helsinki, Helsinki, Finland; f Department of Medicine, Central Finland Health Care District, Jyväskylä, Finland; g Faculty of Education and Psychology, University of Jyväskylä, Jyväskylä, Finland; h Obesity Research Unit, Research Program for Clinical and Molecular Medicine, Faculty of Medicine, University of Helsinki, Helsinki, Finland; i Department of Clinical Microbiology, Turku University Hospital, Turku, Finland; j Helsinki University Hospital, Population Health Research, Finnish Institute for Health and Welfare, Helsinki and Oulu, Finland; k Institute of Clinical Medicine, Department of Medicine, University of Eastern Finland, Kuopio, Finland; l HealthyWeightHub, Abdominal Center, Helsinki University Hospital, University of Helsinki, Helsinki, Finland; Washington University in St. Louis; Cornell University

**Keywords:** gut microbiota, insulin resistance, liver fat, metabolomics

## Abstract

Numerous studies have described specific metabolites as biomarkers of severe liver diseases, but very few have measured gut microbiota (GM)-produced metabolites in fatty liver disease. We aimed at finding GM signatures and metabolite markers in plasma and feces related to high liver fat content. Based on imaging, we divided study participants into low (<5%, LF, *n *=* *25) and high (>5%, HF, *n *=* *39) liver fat groups. Fecal (LF *n *=* *14, HF *n *=* *25) and plasma (LF *n *=* *11, HF *n *=* *7) metabolomes of subsets of participants were studied using liquid chromatography/high resolution mass spectrometry. The GM were analyzed using 16S rRNA gene sequencing. Additionally, blood clinical variables and diet were studied. Dyslipidemia, higher liver enzymes and insulin resistance characterized the HF group. No major differences in diet were found between the groups. In the GM, the HF group had lower abundance of *Bacteroides* and Prevotellaceae NK3B31 group than the LF group after adjusting for metformin use or obesity. In feces, the HF group had higher levels of lysine and histidine degradation products, while 6-hydroxybetatestosterone (metabolized by CYP3A4) was low. Higher plasma levels of caffeine and its metabolites in the HF group indicate that the activity of hepatic CYP1A2 was lower than in the LF group. Our results suggest, that low fecal Prevotellaceae NK3B31 and *Bacteroides* abundance, and increased lysine and histidine degradation may serve as GM biomarkers of high liver fat. Altered plasma caffeine metabolites and lowered testosterone metabolism may specify decreased CYP activities, and their potential utility, as biomarkers of fatty liver disease.

## INTRODUCTION

Nonalcoholic fatty liver disease (NAFLD), with a worldwide prevalence of up to 40% in adult population, is an acutely increasing burden on public health (1). NAFLD is characterized by hepatic steatosis, i.e., excess (>5%) fat accumulation in hepatocytes (2). Obesity and insulin resistance are associated with NAFLD (3). NAFLD is shown to be present in 40 to 80% of patients with type 2 diabetes (1, 4, 5), and in 30 to 90% of patients with obesity (6). Dietary factors, such as high fructose intake contribute to the development of NAFLD (7). In addition, defective hepatic CYP enzyme activities are well known molecular players in NAFLD (8).

The gut microbiota (GM) referring to the ~100 trillion microbes inhabiting the gastrointestinal tract (9) contribute to the pathophysiology of NAFLD. We have found that in humans high hepatic fat content is linked to lower abundance of Faecalibacterium prausnitzii (10), and further, in a mouse model we have been able to ameliorate NAFLD by administering *F. prausnitzii* (11). One previous study reported that NAFLD patients have lower abundance of Bacteroidetes (12), and another lower abundance of *Bacteroides* (13). Contrarily, others have shown higher abundance of *Bacteroides* in NAFLD (14, 15).

It is increasingly accepted that the abundance of certain members of the GM affect human physiology by processing the ingested food to bioactive metabolites. These molecules act as signaling messengers after absorption in extraintestinal tissues (16), including the liver, which is the first target organ that metabolizes them (17). Despite the importance of the gut-liver axis, studies describing the importance of fecal metabolites in human NAFLD are surprisingly scarce. One study reported that the levels of fecal bile acids and propionate are increased in NAFLD patients depending on the severity of hepatic fibrosis (18). Another study has shown elevated fecal propionate in NAFLD patients, accompanied with higher levels of acetate and butyrate (19). Contrarily, lower levels of acetate are found in obese pediatric NAFLD patients, while propionate is unaffected (20). These results suggest the role of GM in NAFLD, but more studies on the fecal metabolome are needed to assess whether short-chain fatty acids or other GM metabolites could be useful diagnostic biomarkers of NAFLD.

Metabolic profiling of plasma and serum samples have been used to study the pathophysiology of NAFLD, and has, for example linked bile acid homeostasis to NAFLD (21, 22). However, most studies have focused on diagnosing advanced hepatic fibrosis or differentiating between nonalcoholic steatohepatitis (NASH) and non-NASH (23, 24), whereas NAFLD have been less studied. One model proposes that NASH, fatty, or healthy liver status could be distinguished based on lipidomics, glycomics, and hormones (25). A few studies have identified serum lipid signatures, which might be used as diagnostic markers for NAFLD (26, 27). In addition, one recent study discovered 11 metabolites and three clinical parameters, which were associated with NAFLD (28).

Because the high prevalence of NAFLD sets diagnostic challenges to the health care system, we examined the GM, fecal and plasma metabolites, and diet as well as clinical parameters of humans with low (LF, <5%) and high liver (HF, >5%) fat content. The aim of this study was to identify new biomarkers of NAFLD that could have potential utility as diagnostic markers of high liver fat content.

## RESULTS

### Anthropometric and clinical variables differed between the liver fat groups.

The HF participants had higher body weight, BMI, waist circumference, and blood pressure than the LF participants (*P* < 0.05 for all, Table 1). The HF group had 24 males and 15 females (61.5% and 38.5%, respectively), and the LF group 11 males and 14 females (44% and 56%, respectively), with no significant differences in gender between the groups.

**TABLE 1:** Anthropometric measurements, liver fat % and blood pressure of the study participants^*a*^

Variable	LF group (*n *=* *25)	HF group (*n *=* *39)	*P* value
Age (yrs)	48 ± 12	51 ± 10	0.345
ht (cm)	171.4 ± 10.1	175.2 ± 9	0.115
wt (kg)	87 ± 17.2	105.3 ± 20.7	**<0.001**
BMI (kg/m2)	29.8 ± 6.3	34.2 ± 5.8	**0.005**
Waist circumference (cm)	101.1 ± 14	117.8 ± 13.9	**<0.001**
BP systolic (mm Hg)	127 ± 18 (*n* = 24)	138 ± 18	**0.042**
BP diastolic (mm Hg)	81 ± 11 (*n* = 24)	87 ± 9	**0.024**
Liver fat %	1.3 ± 1.3	17.2 ± 10	**<0.001**

a The data are presented as mean ± SD. LF < 5% fat, HF > 5% fat in liver. Bolded *P* values indicate statistically significant difference between the groups. BMI, body mass index; BP, blood pressure; LF, low fiver fat; HF, high liver fat.

The HF participants had ~2-times higher alanine aminotransferase (ALT), aspartate aminotransferase (AST) and gamma-glutamytransferase (GGT) than the LF participants (*P* < 0.001 for all, Table 2), yet only ALT was over the reference range. Compared to the LF, the HF group had ~19% lower HDL (*P* = 0.007) and ~2-times higher triglycerides (*P* < 0.001), and the latter was over the reference range (Table 2). At fasting, oral glucose tolerance test (OGTT) showed ~7% higher levels of glucose (*P* = 0.017) and insulin (<0.001) in the HF group compared to the LF group. Consequently, the HOMA-IR was higher in the HF group (*P* < 0.001), showing significant insulin resistance (Table 2).

**TABLE 2:** Serum clinical variables measured using clinical chemistry^*a*^

Variable	Reference range	LF group (*n *=* *25)	HF group(*n *=* *39)	*P* value
ALT (U/L)	male < 50, female < 35	25.3 ± 11	54 ± 41.1	**<0.001**
AST (U/L)	15–45	23.5 ± 4.3	42.6 ± 35	**<0.001**
GGT(U/L)	<61	27.1 ± 17.2	53.8 ± 32.5	**<0.001**
Cholesterol (mmol/L)	<5	5.2 ± 1.4	5.5 ± 1.3	0.395
HDL (mmol/L)	male > 1, female > 1.2	1.6 ± 0.5	1.3 ± 0.3	**0.007**
LDL (mmol/L)	<3	3 ± 0.6	3.3 ± 0.9	0.282
Trigly (mmol/L)	<1.7	1.1 ± 0.5	2 ± 1.4	**<0.001**
Glucose (mmol/L)^*b*^	4–6	5.7 ± 0.5	6.1 ± 0.7	**0.017**
Insulin (iU/L)^*b*^	2–20	9.1 ± 9.0	19.4 ± 13.1	**<0.001**
HOMA-IR^*b*^	IS < 1, early IR < 1.9, significant IR > 2.9	2.3 ± 2.2	5.4 ± 3.8	**<0.001**

a The data are presented as mean ± SD. LF < 5% fat, HF > 5% fat in liver. Bolded *P* values indicate statistically significant difference between the groups. The reference ranges are based on values obtained with standard methods in the accredited laboratory HUSLAB, Finland (www.huslab.fi/ohjekirja). ALT, alanine aminotransferase; AST, aspartate aminotransferase; GGT, gamma-glutamyl transferase; HDL, high-density lipoprotein; LDL, low-density lipoprotein; Trigly, triglycerides; HOMA-IR, Homeostatic Model Assessment of Insulin Resistance; IS, insulin sensitive; IR, insulin resistance; LF, low fiver fat; HF, high liver fat.

b LF *n *=* *23, HF *n *=* *32. The lower number of participants in OGTT is because it was not done for diabetic participants. HOMA-IR was calculated as fasting insulin (mU/L) x fasting glucose (mmol/L)/22.5.

### The HF participants had lower GM diversity and different GM composition than the LF group.

**FIG 1:**
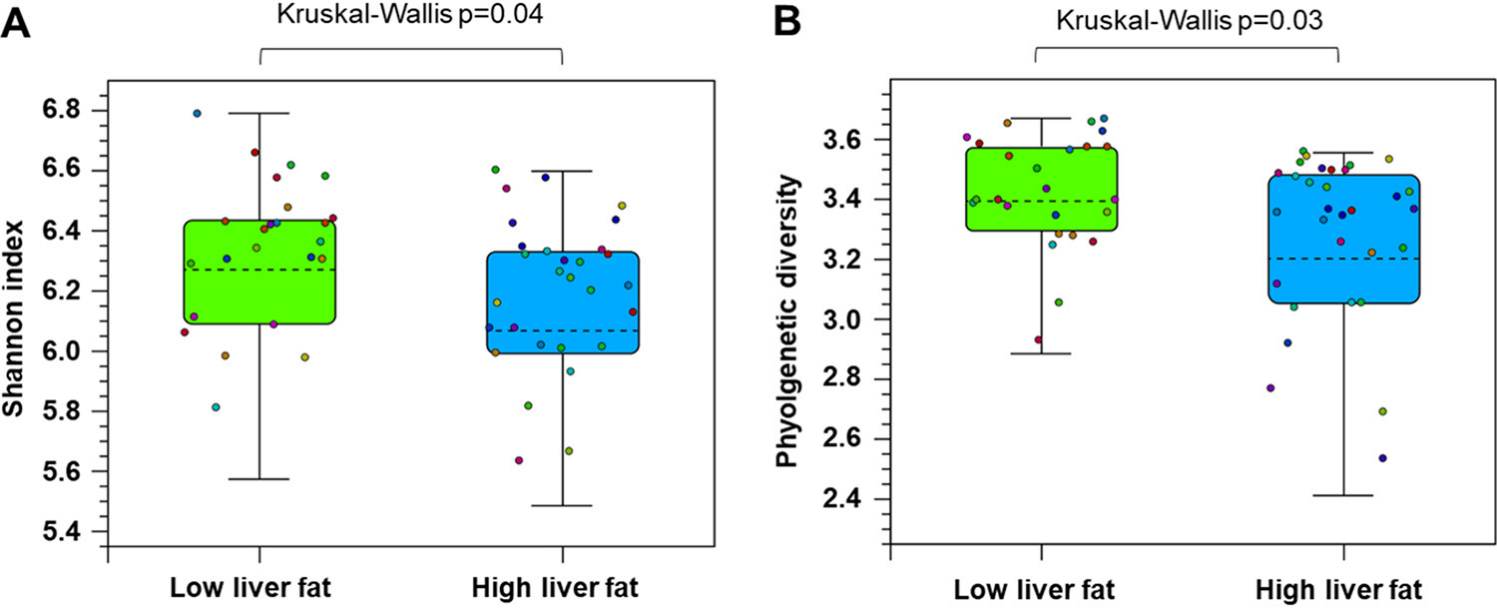
The diversity of the gut microbiota in the low (*n *=* *25) and high (*n *=* *37) liver fat groups. (A) Shannon index, a measure of alpha-diversity indicating species diversity. (B) Phylogenetic diversity. The data are shown as mean ± 95 CI, with the dots indicating diversity in individual samples. Statistical differences between the groups are shown above the panels (Kruskal-Wallis test).

By analyzing the GM alpha-diversity, we found that the HF group had lower Shannon index (i.e., species diversity) (*P* = 0.04, Fig. 1A), while no differences were seen in Chao1 (i.e., species richness, Fig. S1A). In addition, the HF group had lower GM phylogenetic diversity than the LF group (*P* = 0.03, Fig. 1B). Bray Curtis distance showed that the groups did not differ in beta-diversity (i.e., interindividual species diversity, Fig. S1B).

10.1128/mbio.02663-22.2 FIG S1 The diversity of the gut microbiota in the low (*n *=* *25) and high (*n *=* *37) liver fat groups. (A) Chao1, a measure of alpha-diversity indicating species richness. The data are shown as mean ± 95 CI, with the dots indicating diversity in individual samples. Statistical differences between the groups are shown above the panel (Kruskal-Wallis test). (B) Principal component (PCo) plot of beta-diversity analyzed by Bray Curtis distance. PERMANOVA for significance testing showed no differences between the groups. Download FIG S1, DOCX file, 0.2 MB. Copyright © 2023 Driuchina et al. 2023 Driuchina et al. https://creativecommons.org/licenses/by/4.0/ This content is distributed under the terms of the Creative Commons Attribution 4.0 International license.

**FIG 2:**
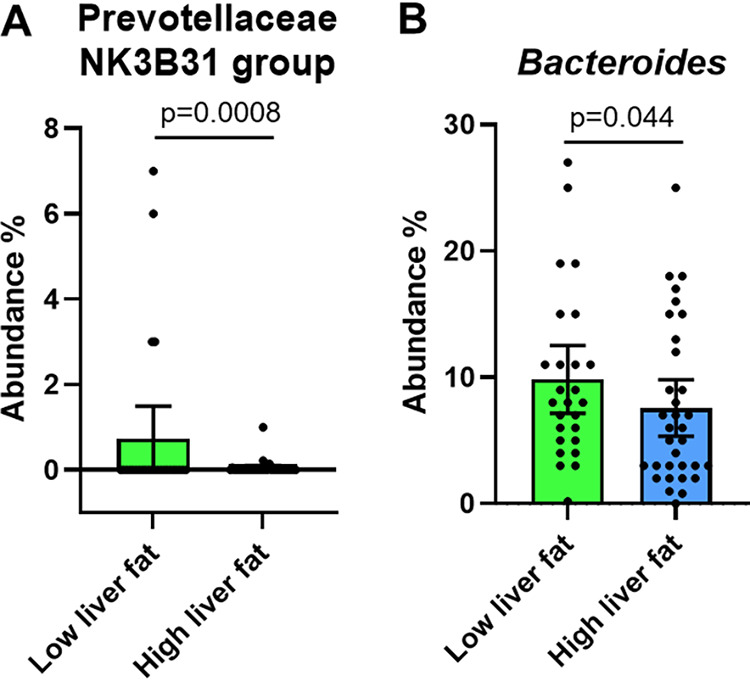
(A and B) The differential abundances of the gut microbiota between the low (*n *=* *25) and high (*n *=* *37) liver fat groups after adjusting for metformin use or obesity. The data are shown as mean ± 95 CI, with the dots indicating abundance % in individual samples. The group differences were analyzed with ANOVA-like test followed by FDR correction.

The average GM composition in the groups is shown in Fig. S2A. In group comparisons, we found differences in the abundance of several taxa (Fig. S2B). However, when adjusted for the use of metformin or obesity, only the difference in *Bacteroides* and Prevotellaceae NK3B31 between the groups remained significant, i.e., the abundance of these was lower in the HF group (Fig. 2A and B).

10.1128/mbio.02663-22.3 FIG S2 A) The average gut microbiota composition in the low (*n *=* *25) and high (*n *=* *37) liver fat groups at phylum, family, and genus level. B) The differences in the composition of the gut microbiota between the low (*n *=* *25) and high (*n *=* *37) liver fat groups without adjusting for the use of metformin or obesity. The data are shown as mean ± 95 CI, with the dots indicating abundance % in individual samples. The group differences were analyzed with ANOVA-like test followed by FDR (false discovery rate) correction. Download FIG S2, DOCX file, 0.4 MB. Copyright © 2023 Driuchina et al. 2023 Driuchina et al. https://creativecommons.org/licenses/by/4.0/ This content is distributed under the terms of the Creative Commons Attribution 4.0 International license.

As diet affects the GM composition, the dietary intakes of macronutrients were analyzed. Notably, we found no significant differences in the energy and nutrient intakes between the groups, except for vitamin E (*P* = 0.035) and sucrose (*P* = 0.038) intakes that were higher in the HF than the LF group (Table S2).

10.1128/mbio.02663-22.8 TABLE S2 Calculated dietary intakes of energy and nutrients per day according the self-reported 3-day food diaries of the study participants with low (*n *=* *25) and high (*n *=* *34) liver fat groups. An average of intakes was calculated from three days, which was used for the analysis. The data are presented as mean ± SD. Download Table S2, DOCX file, 0.02 MB. Copyright © 2023 Driuchina et al. 2023 Driuchina et al. https://creativecommons.org/licenses/by/4.0/ This content is distributed under the terms of the Creative Commons Attribution 4.0 International license.

### The fecal metabolome of the HF group was characterized by high levels of lysine and histidine degradation products.

**FIG 3:**
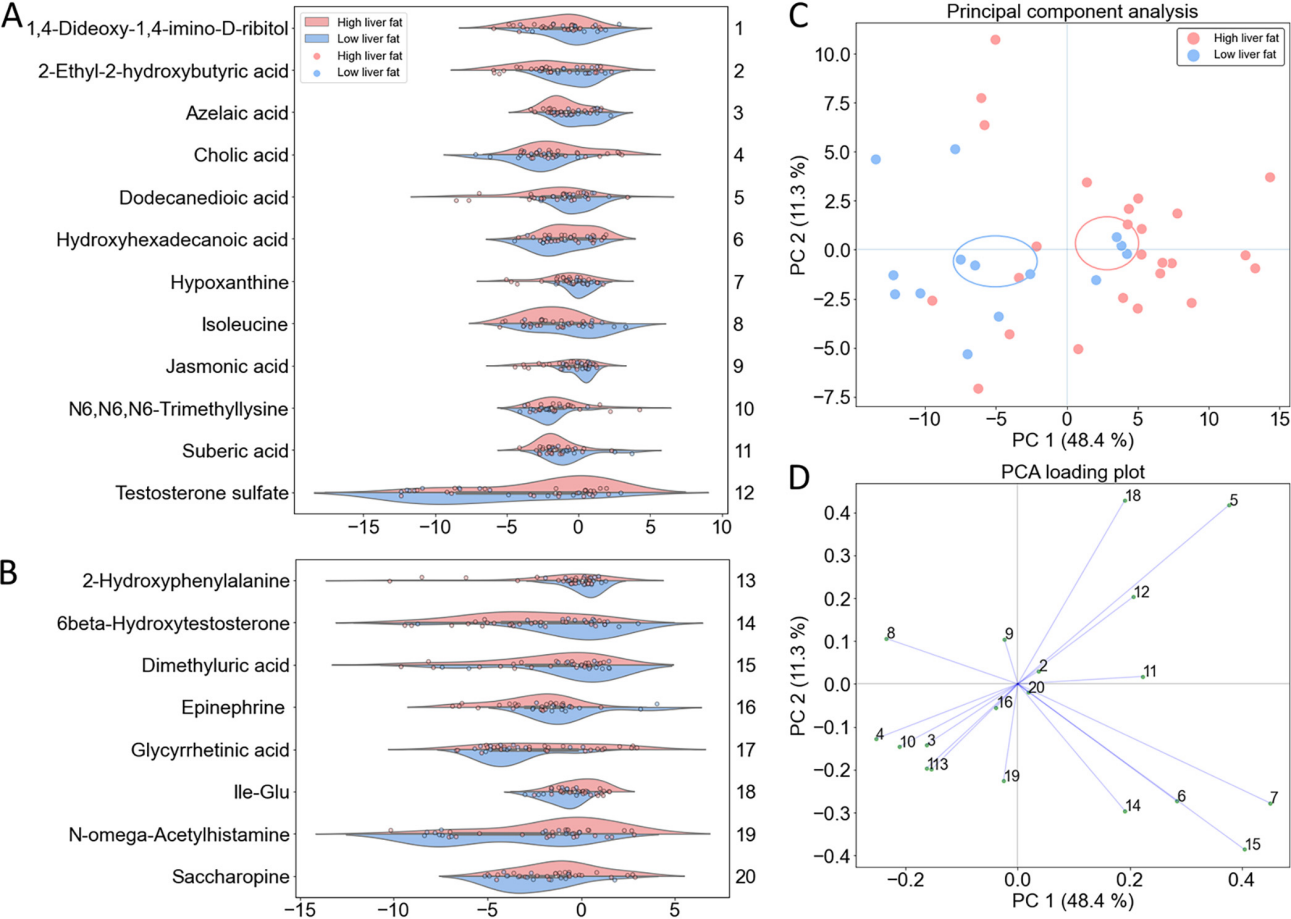
Visualization of the fecal metabolites that (A) differed between the low (*n *=* *14) and high (*n *=* *25) liver fat groups without including confounding factors in the analysis, and (B) differed between the groups after adjusting for body weight and metformin use. The *x* axes indicate log_2-fold_ differences. The group differences were analyzed using Mann-Whitney U-test. Features with a significant difference *P* < 0.05 and fold difference of ≥2 were considered of interest. (C) Principal-component analysis (PCA) of the differing metabolites without adjustment. Circles denote a 95% CI of the scores in each dimension. (D) PCA loading plot of the differing metabolites showing covariances of the variables with the principal components.

We found that the levels of 19 metabolites in feces differed between the groups (Wilcoxon rank-sum *P* < 0.05 and ≥2-fold change, Fig. 3, Table S3A). Identification of the metabolites is presented in Table S1A, and their correlation networks in Fig. S3. Levels of seven metabolites were higher in the HF group varying from 2– to 20-fold compared to LF, and the levels of 12 metabolites in the HF group were 0.16 to 0.50-fold lower from the levels of LF group (Fig. 3A and B, Table S3A). When adjusting for body weight and metformin use, seven metabolites remained significantly different, and the difference in glycyrrhetinic acid became significant (Fig. 3B). The level of testosterone metabolism product, 6-betahydroxytestosterone was very low in the HF group compared to the LF group despite that the HF group included more males. The histidine degradation product N-omega-acetylhistamine, and lysine degradation product saccharopine were higher in the HF group than in the LF group.

10.1128/mbio.02663-22.4 FIG S3 Correlation network community analysis of the fecal metabolites. On the left, a Pearson correlation matrix was computed for all significantly altered fecal metabolites. Metabolite correlations with a Pearson coefficient ≥0.6 and a *P*-value <0.05 were then subject to the Girvan-Newman algorithm to identify highly connected subgraphs. This analysis revealed four network “communities” represented by the four colors. On the right, the fecal metabolites in each of the four groups along with the associated Human Metabolome Database number and description are shown. Download FIG S3, DOCX file, 0.2 MB. Copyright © 2023 Driuchina et al. 2023 Driuchina et al. https://creativecommons.org/licenses/by/4.0/ This content is distributed under the terms of the Creative Commons Attribution 4.0 International license.

10.1128/mbio.02663-22.7 TABLE S1 Identification information of the metabolites in feces and plasma. A) Fecal metabolites and their identification information. All samples were analyzed using two different chromatographic techniques, *i.e*., reversed phase (RP) and hydrophilic interaction chromatography (HILIC). Data were acquired in both electrospray ionization (ESI) polarities, i.e., ESI positive (ESI+) and ESI negative (ESI−). B) Plasma metabolites and their identification information. All samples were analyzed using two different chromatographic techniques, *i.e*., reversed phase (RP) and hydrophilic interaction chromatography (HILIC). Data were acquired in both electrospray ionization (ESI) polarities, i.e., ESI positive (ESI+) and ESI negative (ESI−). Download Table S1, DOCX file, 0.03 MB. Copyright © 2023 Driuchina et al. 2023 Driuchina et al. https://creativecommons.org/licenses/by/4.0/ This content is distributed under the terms of the Creative Commons Attribution 4.0 International license.

10.1128/mbio.02663-22.9 TABLE S3 Fecal metabolites and liver fat. A) The fecal metabolites that differed between the low (*n *=* *14) and high (*n *=* *25) liver fat groups, and their fold changes between the groups. B) Associations between the fecal metabolites and liver fat percentage. The first two columns show the Spearman correlation coefficient and *P*-value without adjusting, respectively. The last two columns show the Spearman correlation coefficient and *P*-value after adjusting for the use of metformin and body weight. Download Table S3, DOCX file, 0.03 MB. Copyright © 2023 Driuchina et al. 2023 Driuchina et al. https://creativecommons.org/licenses/by/4.0/ This content is distributed under the terms of the Creative Commons Attribution 4.0 International license.

**FIG 4:**
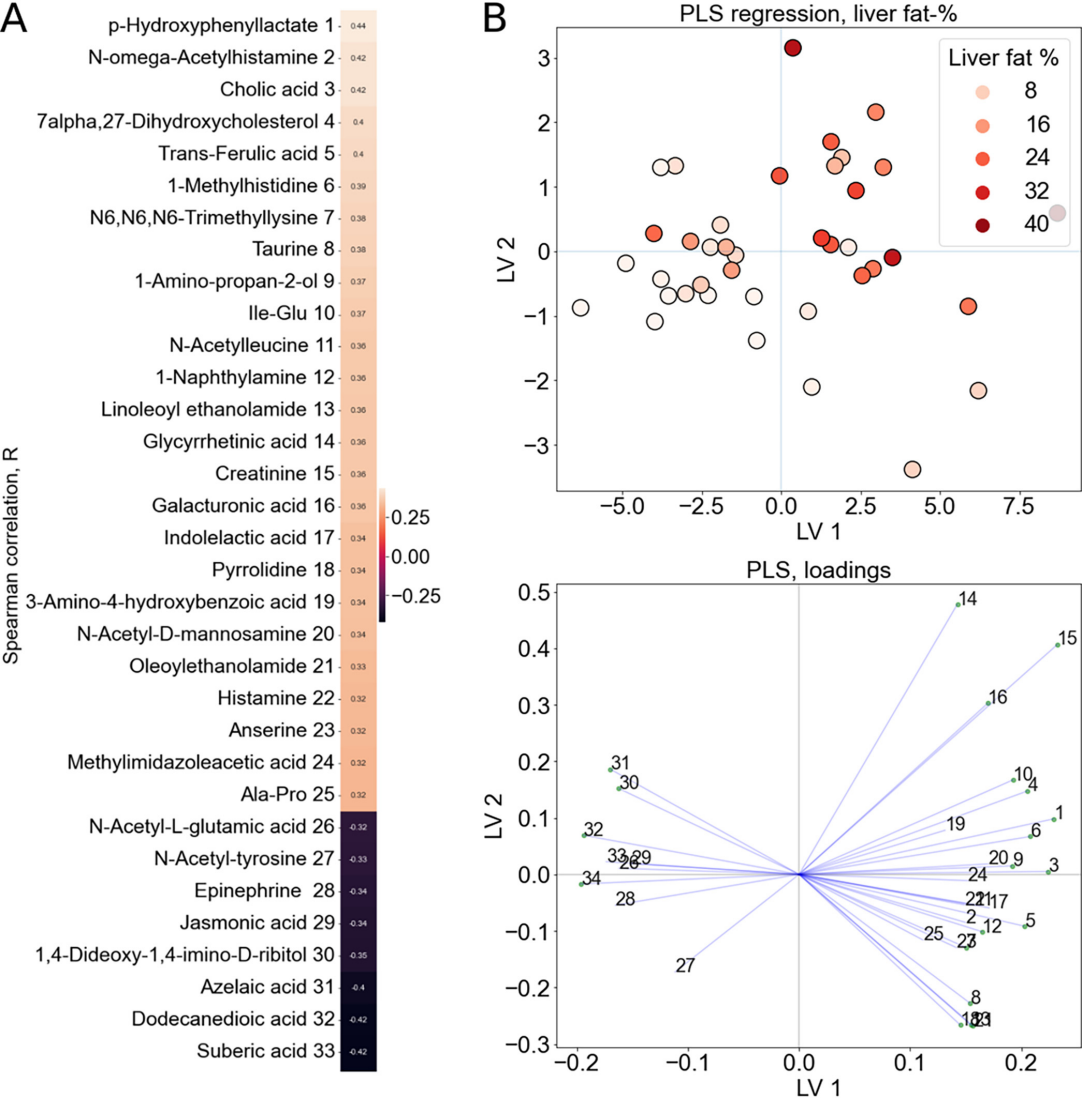
The features with significant Spearman correlations (*P* < 0.05) between the fecal metabolites and liver fat % visualized with (A) heatmap and (B) partial least-squares (PLS) plots and loadings. The color in the heatmap corresponds to the correlation coefficient between the metabolites and liver fat % as shown on the scale right side of the heatmap. LV, latent variable.

Next, we analyzed the associations between the fecal metabolites and liver fat % (Fig. 4A and B, Table S3B). Cholic acid and its precursor 7-a,27-dihydroxycholesterol increased along with liver fat %. In addition, histidine metabolism products urocanic acid, methylhistidine, histamine, anserine, N-acetylhistamine, and methylimidazole acetic acid associated positively with liver fat %. The associations of the GM with fecal metabolites are visualized in Fig. S4.

10.1128/mbio.02663-22.5 FIG S4 Visualization of the associations between the gut microbiota genera and fecal metabolites with heatmaps in (A) low liver fat group (*n *=* *25), and (B) high liver fat group (*n *=* *37). Spearman correlation significance *, <0.05; **, <0.01. Download FIG S4, DOCX file, 0.5 MB. Copyright © 2023 Driuchina et al. 2023 Driuchina et al. https://creativecommons.org/licenses/by/4.0/ This content is distributed under the terms of the Creative Commons Attribution 4.0 International license.

### The HF group was characterized by higher plasma levels of caffeine metabolites, primary and secondary bile acids suggesting altered hepatic CYP activities.

**FIG 5:**
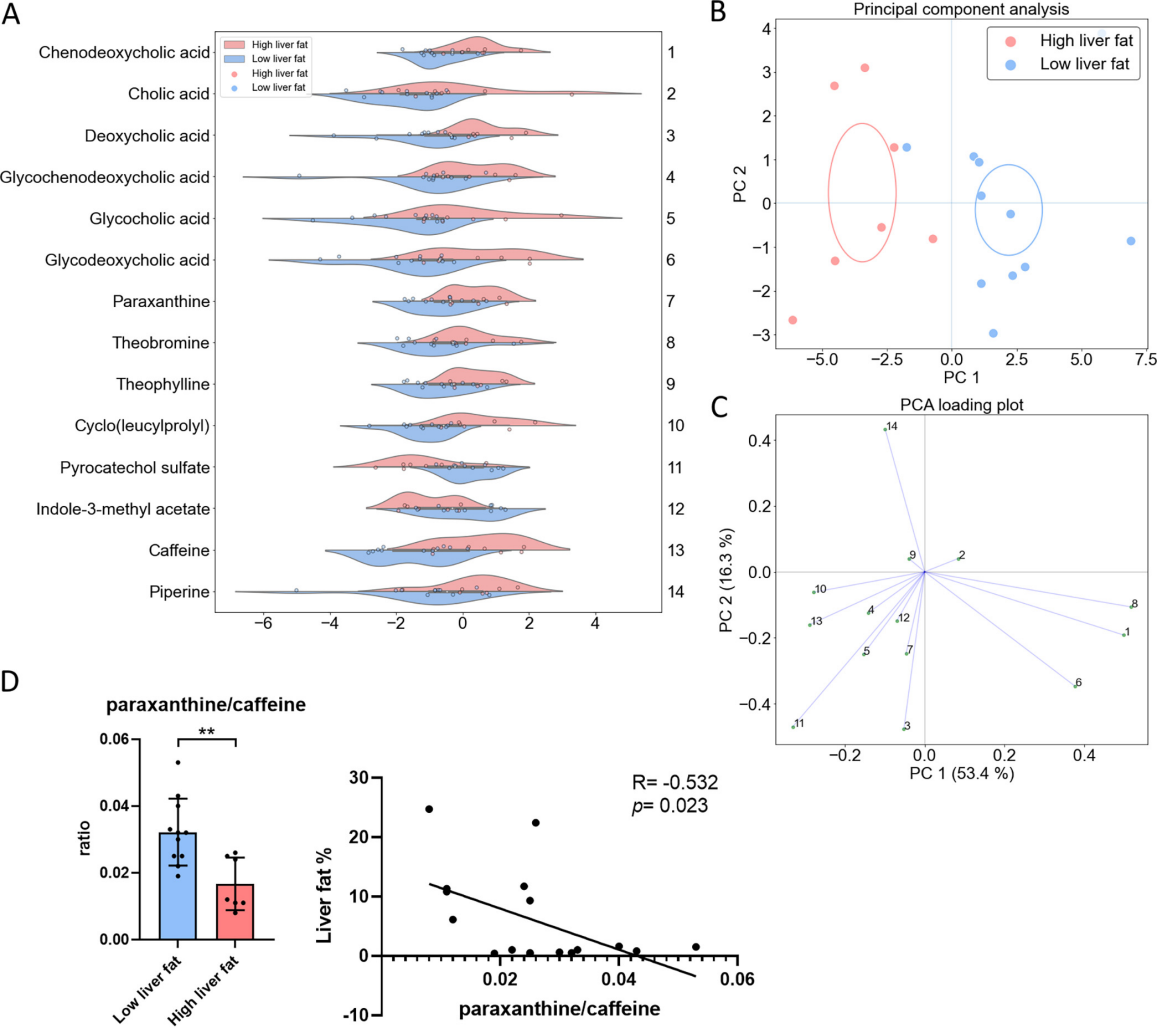
(A) Visualization of the plasma metabolites that differed between the low (*n* = 11) and high (*n* = 7) liver fat groups. The *X* axis indicates log_2_ fold differences. The group differences were analyzed using Mann-Whitney U-test. Features with a significant difference *P* < 0.05 and fold change of ≥ 2 were considered of interest. (B) Principal component analysis (PCA) of the differing metabolites and (C) PCA loading plot of the differing metabolites, showing covariances of the variables with the principal components. (D) Paraxanthine/caffeine ratio, indicative of CYP1A2 activity and its association with liver fat %. In the graph, the data are presented as mean ± SD, and the dots indicate individual data points. The group differences were analyzed with *t*-test and the association with Spearman correlation. **, *P* < 0.01.

We found that 14 plasma metabolites differed between the groups (Wilcoxon rank-sum *P* < 0.05 and ≥2-fold change, Table S4A & Fig. 5). The identification of the metabolites is presented in Table S1B. The levels of 12 metabolites were 2- to 4.3-fold higher in the HF group compared to the LF group. The HF group had higher plasma levels of several bile acids and their glycine conjugates, which are outcome of either hepatic CYP7A1 and metabolized by other hepatic CYPs or higher reabsorption of bile acids. However, we did not find difference in the downstream product of CYP7A1, 7a-hydroxy-4-cholestelen-3-one, between the groups (fold change value HF/LF 0.67 in the plasma and 2.08 in the feces). Caffeine and its metabolites paraxanthine, theophylline, and theobromine as well as cyclo(leucylprolyl), were also higher in the HF group than in the LF group. Notably, there was no difference in coffee intake between the groups (Table S2). To estimate the activity of caffeine-metabolizing CYP1A2, we determined the ratio of paraxanthine/caffeine (29), which was ~2-fold lower in the HF group than in the LF group (*P* = 0.002, Fig. 5D). Further, the ratio associated negatively with liver fat % (R= −0.532, *P* = 0.023, Fig. 5D).

10.1128/mbio.02663-22.10 TABLE S4 Plasma metabolites and liver fat. A) The plasma metabolites that differed between low (*n *=* *11) and high (*n *=* *7) liver fat groups, and their fold changes between the groups. The second column shows the *P*-value without adjusting, and the third the *P*-value after adjusting to body weight. In these study groups there were no participants using metformin, and thus, the use of metformin was not used as covariate as it was in the fecal metabolite analyses. B) Associations between the plasma metabolites and liver fat percentage. The first two columns show the Spearman correlation coefficient and *P*-value without adjusting, respectively. The last two columns show the Spearman correlation coefficient and *P*-value after adjusting for the body weight. In this study group there were no participants using metformin, and, thus the use of metformin was not used as covariate as it was in the fecal metabolite analyses. Download Table S4, DOCX file, 0.03 MB. Copyright © 2023 Driuchina et al. 2023 Driuchina et al. https://creativecommons.org/licenses/by/4.0/ This content is distributed under the terms of the Creative Commons Attribution 4.0 International license.

**FIG 6:**
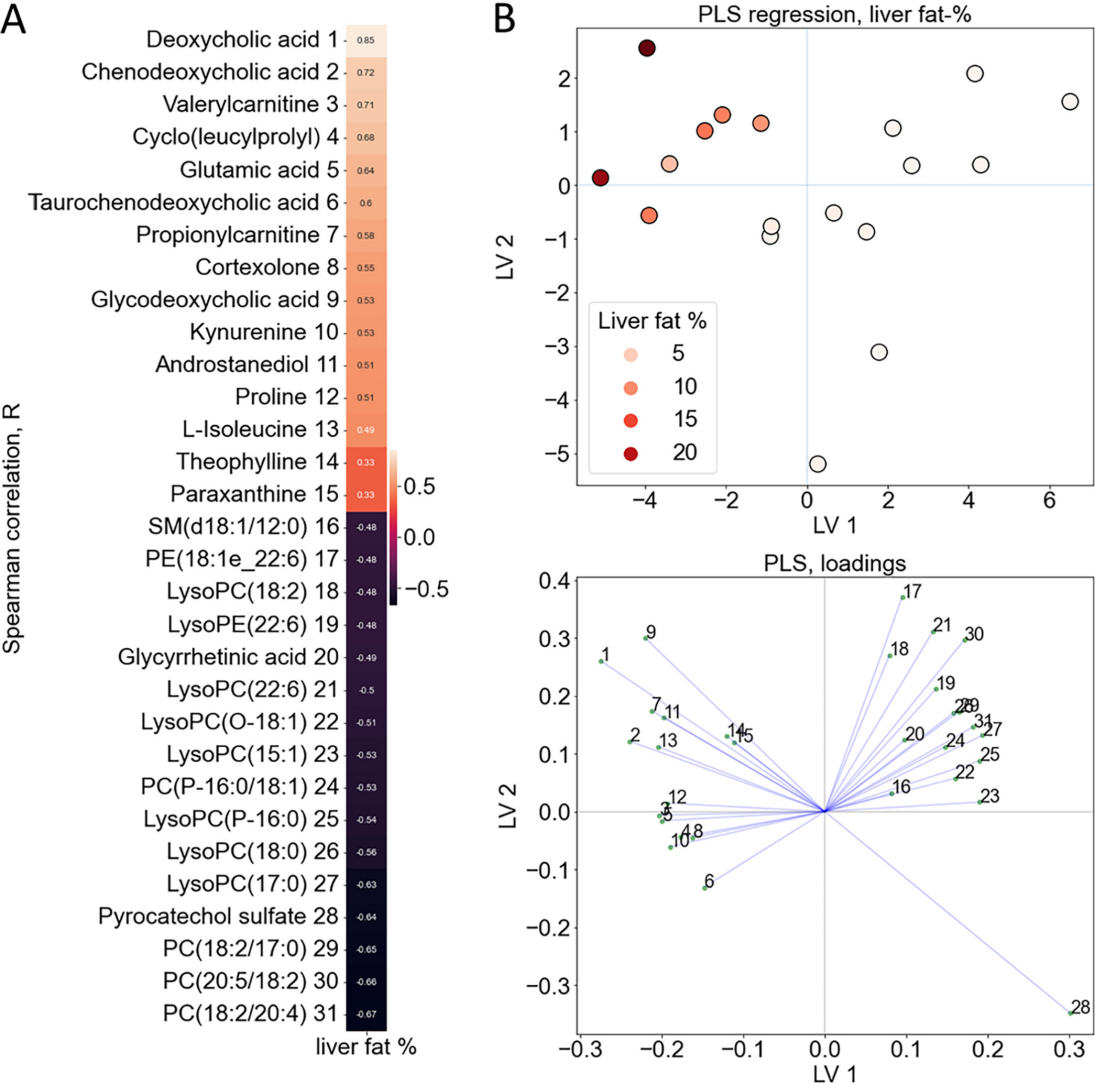
The features with significant Spearman correlations (*P* < 0.05) between the plasma metabolites and liver fat % visualized with (A) heatmap and (B) partial least-squares (PLS) plot (R^2^ = 0.74) and loadings. The color in the heatmap corresponds to the correlation coefficient between the metabolites and liver fat % as shown on the scale right side of the heatmap. LV, latent variable.

We also analyzed the associations between plasma metabolites and liver fat %. Bile acids, their conjugates, cyclo(leucylprolyl) and amino acids proline, isoleucine and glutamic acid associated positively with liver fat %, while several phospholipids associated negatively with it (Fig. 6, Table S4B).

To further understand the metabolic derangements, a correlation-based network community analysis was carried out using the Girvan-Newman algorithm (30), where the metabolites are the network nodes and the connections between the nodes are the Pearson correlations. The network community analysis diagram was generated combining all 38 significant plasma metabolites. The algorithm detected four distinct communities (Fig. S5). The first community included pyrocatechol sulfate and indole-3-methyl acetate. The second community was composed of primary and secondary bile acids as well as two short-chain acylcarnitines. The third community was composed of diet-related metabolites, especially of caffeine metabolism. The last community was composed entirely of lipids, including lysophosphocholines and phosphocholines.

10.1128/mbio.02663-22.6 FIG S5 Correlation network community analysis of the plasma serum metabolites. On the left, a Pearson correlation matrix was computed for all significantly altered serum metabolites. Metabolite correlations with a Pearson coefficient ≥0.6 and a *P*-value <0.05 were then subject to the Girvan-Newman algorithm to identify highly connected subgraphs. This analysis revealed four network “communities” represented by the four colors. On the right, the plasma metabolites in each of the four groups along with the associated Human Metabolome Database number and description are shown. Download FIG S5, DOCX file, 0.1 MB. Copyright © 2023 Driuchina et al. 2023 Driuchina et al. https://creativecommons.org/licenses/by/4.0/ This content is distributed under the terms of the Creative Commons Attribution 4.0 International license.

## DISCUSSION

Because the high prevalence of NAFLD sets diagnostic challenges to health care, we examined the GM, fecal and plasma metabolites as well as clinical parameters of humans with low (LF, <5%) and high liver (HF, >5%) fat content in this study. We identified new potential microbial and plasma metabolic biomarkers of fatty liver disease.

Metabolically, especially higher levels of triglycerides and significant insulin resistance in the HF group suggest their utility as biomarkers of a fatty liver disease without biopsy specimen proven NAFLD. This is supported by earlier studies showing that dyslipidemia (31, 32), and the pathogenesis of insulin resistance (33–35) associated with NAFLD. Our previous study also showed insulin resistance in humans with high liver fat content (>5% based on MRI) (10).

We found that the GM diversity was lower in the HF group, which is consistent, e.g., with the study by Astbury et al. showing lower GM diversity in NAFLD patients (36). When adjusting for obesity or the use of metformin, both of which are known to affect GM (37), the HF group had lower abundance of Prevotellaceae NK3B31 than the LF group. Consistently, we have shown that when a rat model of NAFLD was supplemented with prebiotic Xylo-oligosaccharides, the abundance of Prevotellacaeae NK3B31 increased along with reduction of liver fat (38). Thus, this genus might be an indicator of hepatic steatosis. Our finding on the lower abundance of *Bacteroides* agrees with a study by Testerman et al. in NAFLD patients (13), while opposite findings also exist (15). However, contrary to our results, Testerman and coworkers found that two GM pathways of histidine degradation were decreased in NAFLD patients (13). In contrast, elevated urinary methylhistidine has been described in NAFLD patients (39). In agreement with the latter, we report here that the fecal level of histidine metabolism product, N-omega-acetylhistamine was markedly increased in the HF group. Further, other products of histidine degradation, such as anserine, positively associated with liver fat.

Of the GM metabolites, we also found that saccharopine, a degradation product of lysine was higher in the HF group compared to the LF. In agreement, Michail et al. found by studying GM metagenomes, that the lysine degradation pathway (lysine=>saccharopine=>acetoacetyl-CoA) was exclusively identified in NAFLD patients and not in healthy individuals (20). Together these findings suggest that increased histidine and lysine degradation by the GM may be HF biomarkers. Furthermore, in the feces of the HF group, 6-hydroxybetatestosterone was reduced. Because there were more males in the HF group than in the LF group, our finding further suggests that higher hepatic fat content could associate with lower activity of testosterone-metabolizing CYP3A4 (40).

We found elevated cyclo(leucylprolyl), glycine-conjugated bile acids and caffeine metabolites in the plasma of the HF participants. Interestingly, a previous study has shown that dipeptides, glycine-conjugated bile acids, and lower coffee intake were associated with the incidence of hepatocellular carcinoma and fatal liver disease in a prospective setting (41). Here, we report that paraxanthine, which is the major primary metabolite of caffeine (42), as well as minor primary metabolites theophylline and theobromine, were elevated in the plasma of HF participants, despite no difference in coffee intake between the groups. The results of our metabolite network analysis further supported these metabolic derangements. While, to our knowledge, elevated caffeine metabolites have not been reported in NAFLD, Holstege et al. have shown that the half-life of these metabolites increases as cirrhosis progresses (43). Whether the elevated plasma caffeine metabolites and cyclic dipeptides could be useful biomarkers in detecting NAFLD warrants further research in larger populations.

Caffeine metabolism is a CYP-dependent process in the liver, and alterations in CYP expression and activity have been described in NAFLD (44). We found that the activity of CYP1A2, as estimated from the increased paraxanthine/caffeine ratio (29), was reduced in the HF participants. Accordingly, CYP1A2 mRNA expression and enzyme activity are shown to decrease along with NAFLD progression from steatosis to NASH (8). Further, a decline in CYP1A2 enzymatic activity with exacerbating fibrosis has been observed (45). CYP1A2 is the most important CYP in metabolism of caffeine (29). Taken together, it seems that the low activity of CYP1A2 could be an early biomarker of NAFLD. In agreement with previous studies (46–49), we observed signs of altered bile acid metabolism in the plasma of the HF group, as increased bile acids levels were found both in feces and plasma. Our results therefore suggest that the activity of bile acids metabolizing CYP7A1 (50) is increased in the HF group. However, we did not find differences in the downstream product of CYP7A1, 7a-hydroxy-4-cholestelen-3-one, between the groups which suggests that CYP7a1 activity is not decreased. This is in agreement with what has been shown in NAFLD and NASH (51).

Our study is not without limitations. The sample sizes for metabolite analyses can be considered rather small, and we could not find similar cohorts to reproduce our findings and to compare our results with others. Because the participants self-collected the fecal samples at their homes, the samples could not be immediately frozen at −80°C. Thus, some microbial metabolism may have occurred during the 1 to 2 days when the samples were frozen at nonstandardized home freezers.

In conclusion, our study suggests that lower Prevotellaceae NK3B31 group and *Bacteroides* abundance as well as increased lysine and histidine degradation in feces could be microbial biomarkers of high liver fat. Further, higher plasma caffeine metabolites and signs of altered testosterone metabolism, suggesting lower hepatic CYP1A2 and CYP3A4 activities may be used as biomarkers of high liver fat. However, to establish the use of these variables as biomarkers in detecting the risk of NAFLD warrants further research in larger populations.

## MATERIALS AND METHODS

### The human study participants.

The study was approved by the Ethics Committee of the Hospital District of Southwest Finland (ETMK 72/2019) and by the Helsinki University Hospital (270/13/03/01/2008). The inclusion criteria were an age <75 years, being overweight (body mass index [BMI] >25), and high waist circumference (>102 cm for males, >88 cm for females). The exclusion criteria were antibiotic treatment 1 month prior to the study, excessive alcohol consumption (>20 g/day for females, 30 g/day for males), inflammatory bowel disease, celiac disease, major eating disorders, cardiovascular diseases, and hypothyroidism. Informed consent was collected from all participants prior to the study.

The participants were recruited by the University of Jyväskylä and the University of Helsinki, Finland, and data were analyzed between 2020 and 2022. The participants were divided into low (LF), and high (HF) liver fat groups based on magnetic resonance imaging (MRI) or magnetic resonance spectroscopy (MRS) quantification of the hepatic triglyceride content. Details of the imaging analyses are provided in Supplementary material (Text S1). Liver fat was measured with MRI for 46 eligible participants and with MRS for eligible 18 participants. Still, there is no consensus on the exact fat % threshold distinguishing between healthy and fatty liver. Based on the literature (2), we considered the participants as having fatty liver disease, when the liver had over 5% fat. Based on the measurements, 39 participants had high (>5% fat, HF) and 25 had low (<5% fat, LF) liver fat. The participants did not have clinical diagnosis of NAFLD, and thus, for clarity we refer to fatty liver disease.

### Oral glucose tolerance test and blood samples.

Oral glucose tolerance test (OGTT) was performed for nondiabetic participants (*n *=* *59) after overnight fasting. Blood samples were collected prior to commencing the OGTT and at 30, 60 and 120 min. Homeostatic Model Assessment for Insulin Resistance (HOMA-IR) was calculated as fasting insulin (mU/L) x fasting glucose (mmol/L)/22.5 (52, 53). Serum AST, ALT, GGT, glucose, insulin and triglycerides were analyzed using standardized clinical methods at HUSLAB laboratories (Helsinki, Finland) or bioanalytical facility of the Faculty of Sport and Health Sciences, University of Jyväskylä, Finland.

### Body composition measurements.

Height was measured using a wall-fixed measuring device, and weight was measured using an electronic scale. BMI was calculated as weight (kg)/height (m)^2^. Waist circumference was measured midway between the lowest rib and the iliac crest twice with a tape measure and the mean value was used for the analyses.

### Fecal sample collection and 16S rRNA gene sequencing.

The participants self-collected the fecal samples, which were home-frozen immediately after collection, brought to laboratory frozen and stored at −80°C. The bacterial DNA was extracted using Stool-kit v2 and semiautomated GenoXtract (Hain Lifescience, Nehren, Germany) accompanied with preceding bead-beating in 0.5/1.0 mm ceramic bead tubes. The 16S rRNA gene was amplified using primers targeting the V3-V4 regions. The protocol is described in Supplementary material (Text S1). 16S rRNA gene sequences were clustered into operational taxonomic units (OTUs) at 97% similarity using CLC Microbial Genomics Package (Qiagen, Hilden, Germany). The rRNA gene sequences were classified using SILVA SSU Reference database (v132, 99%).

### Analyses of fecal and plasma metabolites from subsets of participants.

Plasma sample preparation for metabolite analyses (LF *n *=* *11, HF *n *=* *7) was done as previously (54). Briefly, samples were thawed on ice and a 100 μL aliquot of plasma was dispensed into a 96-well filter plate (Captiva ND, 0.2 μm PP, Agilent Technologies) containing 400 μL of ice-cold acetonitrile. Samples were mixed to thoroughly precipitate plasma proteins, and then centrifuged 700 × *g* for 5 min at 4°C and the supernatants were collected to a 96-well storage plate and stored at 10°C.

The fecal samples (LF *n *=* *14, HF *n *=* *25) were suspended in phosphate-buffered saline with a ratio of 1:5 (w:v), vortexed for 10 min at full speed and centrifuged 700 × *g* for 5 min at 4°C. An aliquot of 100 μL of the fecal extract was combined with 500 μL of ice-cold methanol, vortexed for 30 sec at full speed and centrifuged 700 × *g* for 5 min at 4°C. The supernatant was transferred to a clean microcentrifuge tube through a 0.2 μm syringe filter.

Nontargeted metabolic profiling was performed at the LC-MS metabolomics center (Biocenter Kuopio, University of Eastern Finland, Finland). The analysis was carried out using an ultra-high performance liquid chromatography (Vanquish Flex UHPLC system, Thermo Scientific, Bremen, Germany, and a 1290 LC system, Agilent Technologies, Waldbronn, Germany) coupled online to a high-resolution mass spectrometry (Q Exactive Focus, Thermo Scientific and 6540 qTOF-MS, Agilent Technologies). All samples were analyzed using two different chromatographic techniques, i.e., reversed phase (RP) and hydrophilic interaction chromatography (HILIC). Data were acquired in both electrospray ionization (ESI) polarities, i.e., ESI positive (ESI+) and ESI negative (ESI−). Data-dependent product ion spectrums (MS2 data) were acquired from pooled quality control (QC) samples at the beginning and end of the analysis for each mode. QC samples were injected in the beginning of the analysis and after every 12 samples. The LC-MS instrument set-ups and data acquisition parameters have been described previously (55). Detailed analyses of the samples and data are described in Supplemental material (Text S1). The identification information on the fecal and plasma metabolites are shown in Tables S1A and B, respectively.

### Diet analyses.

The diet was analyzed from 3-day self-reported food diaries (2 weekdays and 1 weekend day) using Aivodiet software (Aivodiet, Flow-team Oy, Oulu, Finland). An average daily intake of energy and nutrients were calculated from the 3 days. Fecal sampling and food diary filling were performed within the same week.

### Statistical analyses.

Statistical analyses of clinical variables and diet were performed using IBM SPSS Statistics 26 (Armonk, NY, USA). Normal distribution of the variables was analyzed using Shapiro Wilk’s test. Differences between the groups were analyzed using independent samples T-test or Mann-Whitney U-test. Statistical significance was set at *P* < 0.05.

Statistical analyses of the GM were performed with CLC Microbial Genomics Package (Qiagen). The group differences in GM alpha-diversity measures and phylogenetic diversity were analyzed with Kruskal-Wallis test. GM beta-diversity analysis was based on Bray-Curtis distance and PERMANOVA (PERmutational Multivariate ANalysis Of Variance) between the groups. GM taxonomic differences between the groups were analyzed with ANOVA-like comparison, followed by Benjamini-Hochberg correction for multiple testing. In the taxonomic comparisons, statistical significance was set at FDR (false discovery rate) *P* < 0.05. The group differences were adjusted for obesity and metformin use.

Statistical analyses of the metabolites were performed using Python 3 and the packages Scipy and Scikit Learn. The raw abundances of the molecular features were log_2_-transformed, the group differences were analyzed using Mann-Whitney U-test and adjusted for obesity and metformin use. Features with a significant difference *P* < 0.05 and fold change of ≥2 between the groups were considered of interest.

### Data availability.

The access to the data is restricted due to personal information protection (General Data Protection Regulation (GDPR) 2016/679 and Directive 95/46/EC). However, it is possible to contact author to ask for a copy of the material. The metadata of the study can be found in https://doi.org/10.17011/jyx/dataset/85068.

10.1128/mbio.02663-22.1 TEXT S1 Methods for Magnetic resonance imaging, Magnetic resonance spectroscopy, Gut microbiota 16S rRNA gene sequencing, Plasma and fecal metabolomic analyses from subsets of study participants. Download Text S1, DOCX file, 0.02 MB. Copyright © 2023 Driuchina et al. 2023 Driuchina et al. https://creativecommons.org/licenses/by/4.0/ This content is distributed under the terms of the Creative Commons Attribution 4.0 International license.
