# Supplementary material for: Identification of Gut Microbial Lysine and Histidine Degradation and CYP-Dependent Metabolites as Biomarkers of Fatty Liver Disease
Source: mBio. 2023 Jan 30;14(1):e02663-22. doi: 10.1128/mbio.02663-22 (PMC9973343; doi:10.1128/mbio.02663-22)
Supplement: FIG S1 [file mbio.02663-22-s0002.docx]

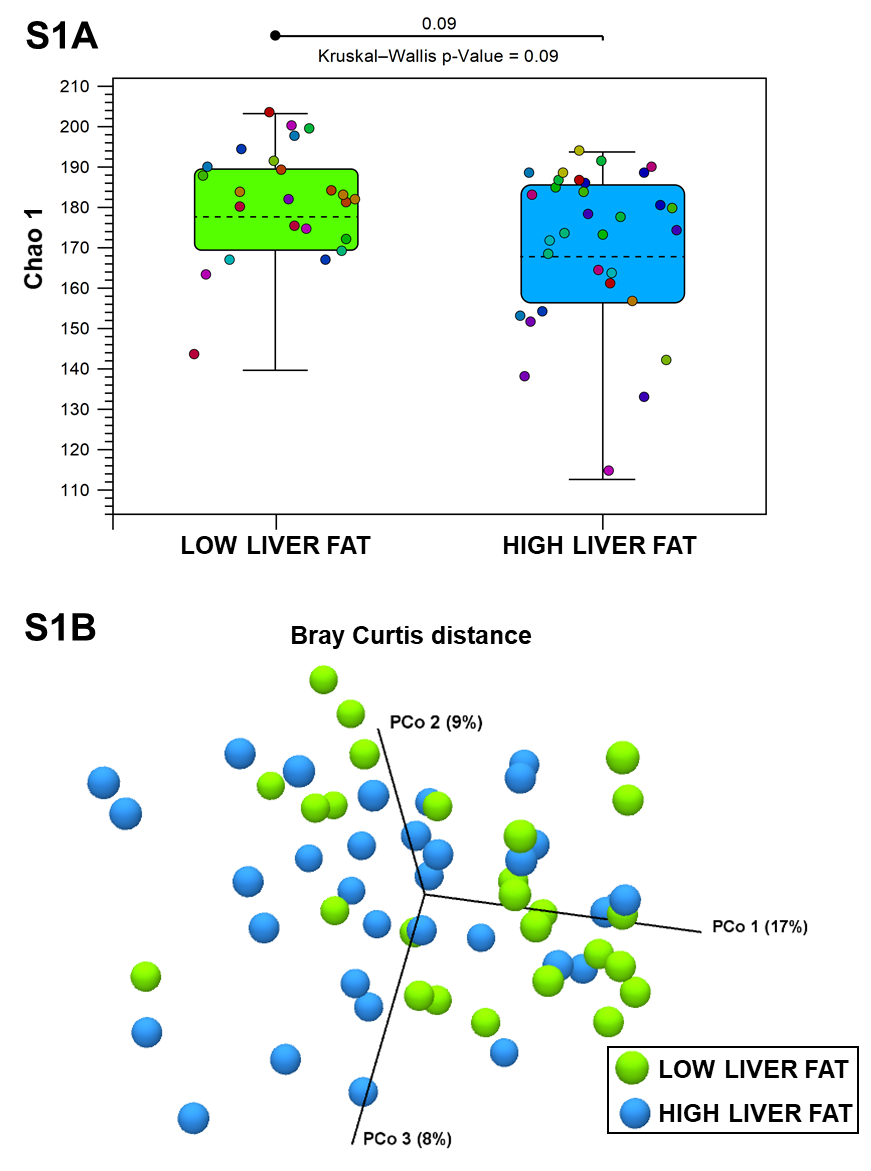


**Fig.** **S1.** The diversity of the gut microbiota in the low (*n*=25) and high (*n*=37) liver fat groups. **(A)** Chao1, a measure of alpha-diversity indicating species richness. The data are shown as mean ± 95 CI, with the dots indicating diversity in individual samples. Statistical differences between the groups are shown above the panel (Kruskal-Wallis test). **(B)** Principal component (PCo) plot of beta-diversity analyzed by Bray Curtis distance. PERMANOVA for significance testing showed no differences between the groups.
