# Supplementary material for: Identification of Gut Microbial Lysine and Histidine Degradation and CYP-Dependent Metabolites as Biomarkers of Fatty Liver Disease
Source: mBio. 2023 Jan 30;14(1):e02663-22. doi: 10.1128/mbio.02663-22 (PMC9973343; doi:10.1128/mbio.02663-22)
Supplement: FIG S2 [file mbio.02663-22-s0003.docx]

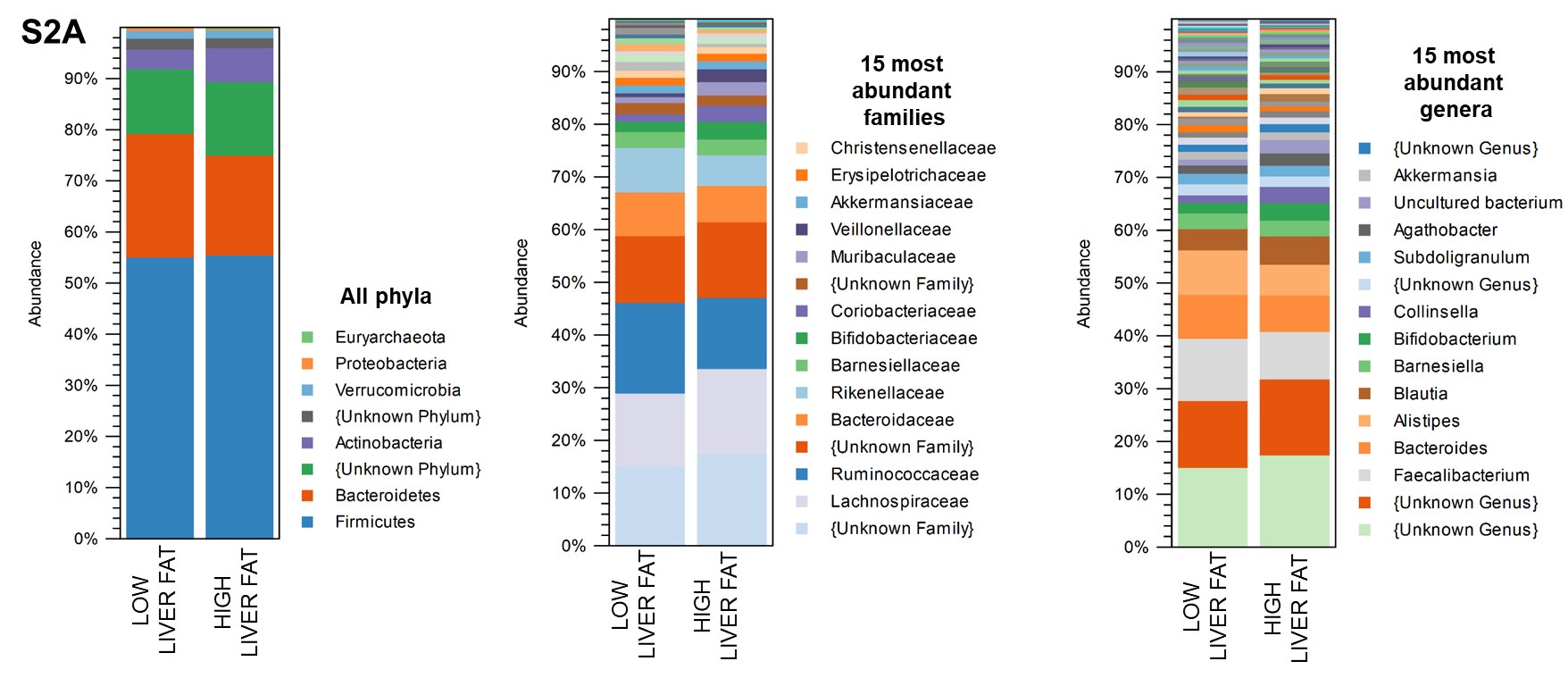


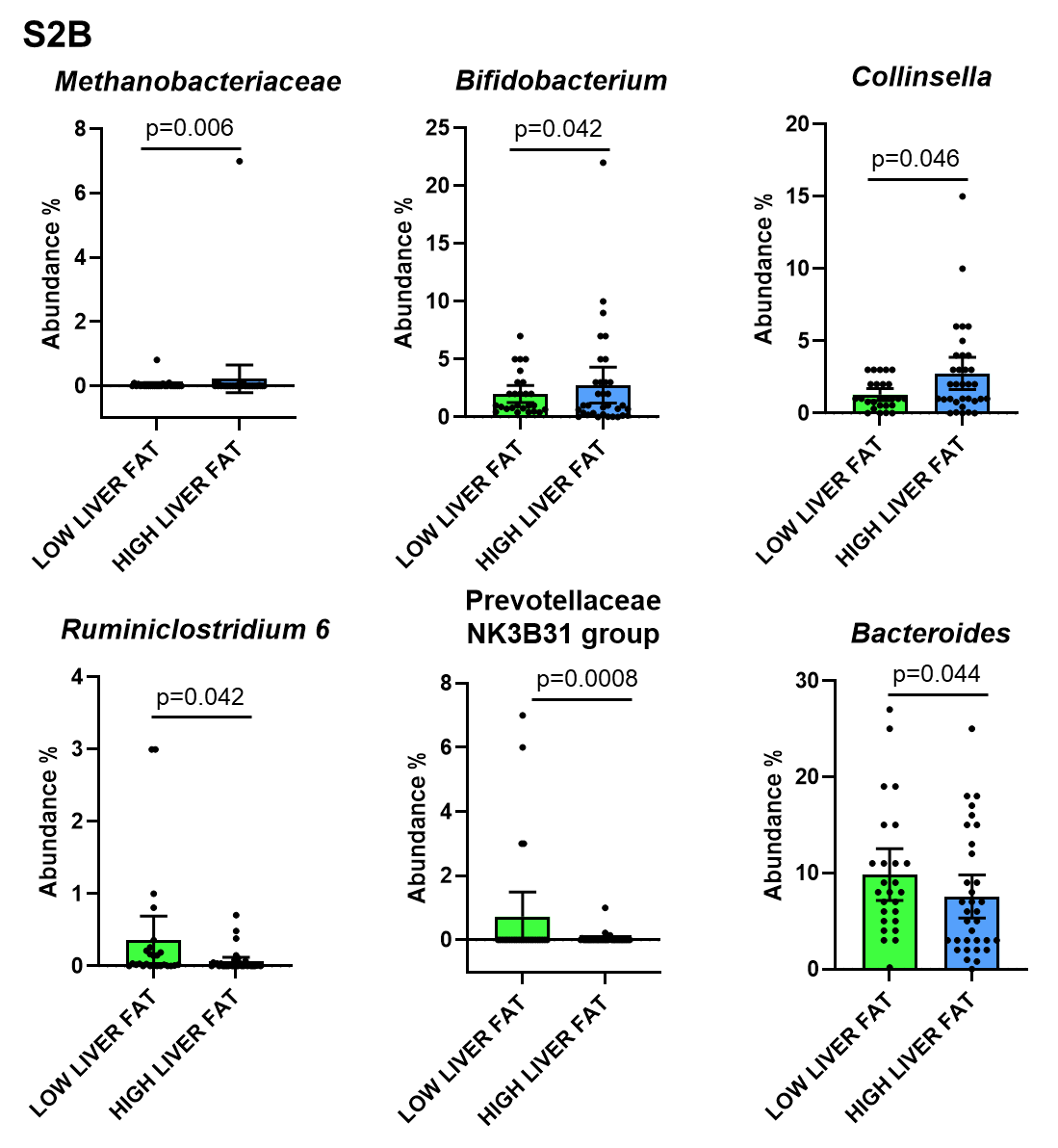


**Fig. S2 A)** The average gut microbiota composition in the low (*n*=25) and high (*n*=37) liver fat groups at phylum, family, and genus level. **B)** The differences in the composition of the gut microbiota between the low (*n*=25) and high (*n*=37) liver fat groups without adjusting for the use of metformin or obesity. The data are shown as mean ± 95 CI, with the dots indicating abundance % in individual samples. The group differences were analyzed with ANOVA-like test followed by FDR (false discovery rate) correction.
