# Supplementary material for: Identification of Gut Microbial Lysine and Histidine Degradation and CYP-Dependent Metabolites as Biomarkers of Fatty Liver Disease
Source: mBio. 2023 Jan 30;14(1):e02663-22. doi: 10.1128/mbio.02663-22 (PMC9973343; doi:10.1128/mbio.02663-22)
Supplement: TABLE S2 [file mbio.02663-22-s0008.docx]

**Table S2.** Calculated dietary intakes of energy and nutrients per day according to the self-reported 3-day food diaries of the study participants with low (*n*=25) and high (*n*=34) liver fat groups. An average of intakes was calculated from three days, which was used for the analysis. The data are presented as mean ± SD.

| **Variable** | **High liver fat**  ***n*=34** | **Low liver fat**  ***n*=25** | **p-value** |
| --- | --- | --- | --- |
| **Energy (kJ)** | 9097 ± 2499 | 9417 ± 2932 | 0.902 |
| **Energy (kcal)** | 2174 ± 597 | 2251 ± 700 | 0.890 |
| **Protein (g)** | 99.5 ± 25.1 | 99.4 ± 32.9 | 0.782 |
| **Carbohydrates (g)** | 194.1 ± 69.6 | 210.8 ± 93.3 | 0.454 |
| **Fat (g)** | 97.9 ± 34.9 | 100.5 ± 41.6 | 0.988 |
| **Alcohol** | 10.2 ± 20.6 | 4.7 ± 8.7 | 0.469 |
| **Coffee (dl/3 days) *** | 5.7 ± 3.7 | 4.4 ± 3.1 | 0.596 |
| **Fatty acids (g)** | 34.5 ± 14.6 | 35.6 ± 19.6 | 0.818 |
| **Monosaturated FA (g)** | 32.7 ± 13.4 | 33.4 ± 13.3 | 0.656 |
| **Polyunsaturated FA (g)** | 15.4 ± 8.1 | 15.4 ± 5.7 | 0.581 |
| **Sucrose (g)** | 27.7 ± 17.3 | 40.7 ± 23.8 | **0.038** |
| **Fiber, total (g)** | 22.5 ± 12.4 | 29.3 ± 25.7 | 0.170 |
| **Vitamin C (mg)** | 167.5 ± 293.5 | 166 ± 205.7 | 0.434 |
| **Thiamine (B1) (mg)** | 1.57 ± 0.71 | 1.47 ± 0.89 | 0.330 |
| **Vitamin E (mg)** | 10.8 ± 5.3 | 15.3 ± 14.5 | **0.035** |
| **Vitamin A (µg)** | 1000 ± 940 | 2646 ± 6106 | 0.172 |
| **Vitamin D (µg)** | 7.8 ± 4.5 | 11.6 ± 12.5 | 0.211 |
| **Riboflavin (B2) (mg)** | 2 ± 1.1 | 2.3 ± 1.4 | 0.505 |
| **Vitamin K (mg)** | 132 ± 95.7 | 142.9 ± 61.8 | 0.163 |
| **Vitamin B6 (mg)** | 2.35 ± 2.4 | 2.57 ± 2.35 | 0.539 |
| **Folate (B9) (µg)** | 262.4 ± 167.1 | 334.2 ± 507.8 | 0.878 |
| **Sodium (mg)** | 3268 ± 1165 | 2830 ± 781 | 0.276 |
| **Magnesium (mg)** | 385.6 ± 123.6 | 417.5 ± 138.9 | 0.434 |
| **Calcium (mg)** | 1092 ± 498 | 1165 ± 437 | 0.390 |
| **Potassium (mg)** | 3768 ± 1082 | 3930 ± 1119 | 0.866 |
| **Iron (mg)** | 14.7 ± 6.2 | 15.1 ± 6.9 | 0.848 |
| **Iodine (µg)** | 230 ± 230.6 | 208.1 ± 65.8 | 0.462 |
| **Phosphorus (mg)** | 1605 ± 430 | 1675 ± 552 | 0.951 |
| **Selenium (µg)** | 81.7 ±28.5 | 85.4 ± 39.7 | 0.927 |
| **Zinc (mg)** | 13.9 ± 4.5 | 14.1 ± 4.7 | 0.951 |
| **Fatty acids, total (g)** | 85.6 ± 32.9 | 87.6 ± 38.2 | 0.890 |
| **Cholesterol (mg)** | 328.7 ± 154.6 | 354.5 ± 187.4 | 0.713 |
| **Fatty acids, trans (g)** | 1.32 ± 0.62 | 1.35 ± 0.64 | 0.878 |
| **Lactose (g)** | 11 ± 10 | 13.3 ± 10.3 | 0.399 |
| **Fiber, total amount (g)** | 20.6 ± 11.5 | 30.8 ± 29.1 | 0.102 |
| **Vitamin B12 (cobalamine) (µg)** | 7.2 ± 4.9 | 15.2 ± 23.5 | 0.452 |
| **Salt (mg)** | 8466 ± 2868 | 7350 ± 2190 | 0.232 |
| **Omega-6 FA (g)** | 11.7 ± 6.7 | 11.6 ± 4.9 | 0.730 |
| **Omega-3 FA (g)** | 3.9 ± 1.3 | 4.1 ± 1.5 | 0.607 |

kJ=kilojoules, kcal=kilocalories. * LF *n*=11, HF *n*=7. The lower number of subjects with calculated coffee intake is because it was calculated only for those participants, who showed perturbations in caffeine metabolism.
