# Supplementary material for: Identification of Gut Microbial Lysine and Histidine Degradation and CYP-Dependent Metabolites as Biomarkers of Fatty Liver Disease
Source: mBio. 2023 Jan 30;14(1):e02663-22. doi: 10.1128/mbio.02663-22 (PMC9973343; doi:10.1128/mbio.02663-22)
Supplement: TABLE S1 [file mbio.02663-22-s0007.docx]

**Table S1.** Identification information of the metabolites in feces and plasma.

**A)** Fecal metabolites and their identification information. All samples were analyzed using two different chromatographic techniques, *i.e*., reversed phase (RP) and hydrophilic interaction chromatography (HILIC). Data were acquired in both electrospray ionization (ESI) polarities *i.e*., ESI positive (ESI+) and ESI negative (ESI-).

| **Metabolite name** | **ID level** | **m/z** | **Ret.time** | **Column, mode** |
| --- | --- | --- | --- | --- |
| 1,4-Dideoxy-1,4-imino-D-ribitol | 2 | 134.0813 | 0.831 | HILIC_ESI+ |
| Glycyrrhetinic acid | 2 | 471.3471 | 10.193 | RP_ESI+ |
| 1-Amino-propan-2-ol | 2 | 76.07641 | 2.669 | HILIC_ ESI+ |
| 1-Methylhistidine | 1 | 170.0925 | 6.326 | HILIC_ ESI+ |
| 1-Naphthylamine | 2 | 144.0808 | 0.95 | HILIC_ ESI+ |
| 2-Ethyl-2-hydroxybutyric acid | 2 | 131.0706 | 2.933 | RP_ESI- |
| 2-Hydroxyphenylalanine | 2 | 182.0812 | 6.133 | RP_ ESI+ |
| 3-Amino-4-hydroxybenzoic acid | 2 | 152.0343 | 1.166 | HILIC_ ESI- |
| 6beta-Hydroxytestosterone | 2 | 305.2111 | 8.124 | RP_ ESI+ |
| 7alpha,27-Dihydroxycholesterol | 2 | 401.3415 | 10.139 | RP_ ESI+ |
| Ala-Pro | 2 | 187.1078 | 0.9 | RP_ ESI+ |
| Anserine | 2 | 241.1289 | 7.13 | HILIC_ ESI+ |
| Azelaic acid | 1 | 211.0942 | 5.883 | RP_ ESI+ |
| Cholic acid | 1 | 407.2801 | 0.646 | HILIC_ ESI- |
| Creatinine | 2 | 114.0665 | 1.248 | HILIC_ ESI+ |
| Dimethyluric acid | 2 | 197.0671 | 2.954 | RP_ ESI+ |
| Dodecanedioic acid | 2 | 229.1445 | 7.738 | RP_ ESI- |
| Epinephrine | 1 | 184.0969 | 2.131 | HILIC_ ESI+ |
| Galacturonic acid | 2 | 193.0342 | 6.42 | HILIC_ ESI- |
| Histamine | 2 | 112.0874 | 5.578 | HILIC_ ESI+ |
| Hydroxyhexadecanoic acid | 2 | 271.2285 | 9.612 | RP_ ESI- |
| Hypoxanthine | 2 | 159.029 | 1.219 | RP_ ESI+ |
| Ile-Glu | 2 | 261.1451 | 1.676 | RP_ ESI+ |
| Indolelactic acid | 1 | 206.0813 | 4.731 | RP_ ESI+ |
| Isoleucine | 2 | 132.1021 | 3.007 | HILIC_ ESI+ |
| Jasmonic acid | 2 | 211.133 | 4.812 | RP_ ESI+ |
| Linoleoyl ethanolamide | 2 | 324.2898 | 10.427 | RP_ ESI+ |
| Methylimidazoleacetic acid | 2 | 141.066 | 3.568 | HILIC_ ESI+ |
| N6,N6,N6-Trimethyllysine | 1 | 189.16 | 6.802 | HILIC_ ESI+ |
| N-Acetyl-D-mannosamine | 2 | 334.0757 | 4.075 | HILIC_ ESI- |
| N-Acetylleucine | 2 | 172.0971 | 0.741 | HILIC_ ESI- |
| N-Acetyl-L-glutamic acid | 1 | 188.0564 | 1.087 | RP_ ESI- |
| N-Acetyl-tyrosine | 2 | 224.0917 | 1.7 | RP_ ESI+ |
| N-omega-Acetylhistamine | 2 | 154.0976 | 2 | HILIC_ ESI+ |
| Oleoylethanolamide | 2 | 326.3054 | 10.724 | RP_ ESI+ |
| p-Hydroxyphenyllactate | 1 | 181.0503 | 2.952 | RP_ ESI- |
| Pyrrolidine | 2 | 72.08154 | 6.573 | HILIC_ ESI+ |
| Saccharopine | 2 | 259.1288 | 1.166 | RP_ ESI+ |
| Suberic acid | 1 | 173.0815 | 4.856 | RP_ ESI- |
| Taurine | 2 | 124.0062 | 5.41 | HILIC_ ESI- |
| Testosterone sulfate | 2 | 367.1591 | 7.46 | RP_ ESI- |
| Trans-Ferulic acid | 1 | 193.0505 | 4.745 | RP_ ESI- |

**B)** Plasma metabolites and their identification information. All samples were analyzed using two different chromatographic techniques, *i.e*., reversed phase (RP) and hydrophilic interaction chromatography (HILIC). Data were acquired in both electrospray ionization (ESI) polarities *i.e*., ESI positive (ESI+) and ESI negative (ESI-).

| **Metabolite name** | **ID level** | **m/z** | **Ret.time** | **Column, mode** |
| --- | --- | --- | --- | --- |
| Androstanediol | 2 | 257.22626 | 10.055 | RP_ ESI+ |
| Caffeine | 1 | 195.08797 | 3.799 | RP_ ESI- |
| Chenodeoxycholic acid | 2 | 391.28561 | 9.273 | RP_ ESI- |
| Cholic acid | 1 | 373.27362 | 9.292 | RP_ ESI+ |
| Corticosterone | 1 | 347.2215 | 7.084 | RP_ ESI+ |
| Cyclo(leucylprolyl) | 2 | 211.14453 | 4.783 | RP_ESI+ |
| Deoxycholic acid | 2 | 391.28552 | 9.914 | RP_ ESI- |
| Glutamic acid | 1 | 148.06036 | 6.543 | HILIC_ ESI+ |
| Glycochenodeoxycholic acid | 1 | 414.30032 | 9.237 | RP_ ESI+ |
| Glycocholic acid | 1 | 448.30569 | 8.607 | RP_ ESI+ |
| Glycodeoxycholic acid | 1 | 448.3071 | 9.396 | RP_ ESI- |
| Glycyrrhetinic acid | 1 | 469.33243 | 10.267 | RP_ ESI- |
| Indole-3-methyl acetate | 1 | 190.08636 | 6.031 | RP_ ESI+ |
| Isoleucine | 1 | 132.10214 | 4.549 | HILIC_ ESI+ |
| Kynurenine | 1 | 209.09274 | 4.369 | HILIC_ ESI+ |
| LysoPC(15:1) | 2 | 524.33521 | 10.674 | RP_ ESI- |
| LysoPC(17:0) | 1 | 510.35532 | 10.512 | RP_ ESI+ |
| LysoPC(18:0) | 1 | 524.37115 | 10.898 | RP_ ESI+ |
| LysoPC(18:2) | 2 | 520.33899 | 10.288 | RP_ ESI+ |
| LysoPC(22:6) | 1 | 568.33966 | 10.16 | RP_ ESI+ |
| LysoPC(O-18:1) | 2 | 552.36713 | 10.844 | RP_ ESI- |
| LysoPC(P-16:0) | 2 | 502.3273 | 10.667 | RP_ ESI+ |
| LysoPE(22:6) | 2 | 524.27826 | 10.187 | RP_ ESI- |
| Paraxanthine | 1 | 179.05716 | 2.841 | RP_ ESI- |
| PC(18:2/17:0) | 2 | 772.58484 | 13.365 | RP_ ESI+ |
| PC(18:2/20:4) | 2 | 850.56097 | 12.756 | RP_ ESI- |
| PC(20:5/18:2) | 2 | 804.55347 | 12.47 | RP_ ESI+ |
| PC(P-16:0/18:1) | 2 | 744.58978 | 14.072 | RP_ ESI+ |
| PE(18:1e_22:6) | 2 | 776.55847 | 14.048 | RP_ ESI+ |
| Piperine | 2 | 286.14365 | 8.379 | RP_ ESI+ |
| Proline | 2 | 116.07175 | 5.143 | HILIC_ ESI+ |
| Propionylcarnitine | 1 | 218.13844 | 1.407 | RP_ ESI+ |
| Pyrocatechol sulfate | 2 | 188.98773 | 0.522 | HILIC_ESI- |
| SM(d18:1/12:0) | 2 | 647.51202 | 11.731 | RP_ ESI+ |
| Taurochenodeoxycholic acid | 2 | 167.0209 | 0.915 | RP_ ESI- |
| Theobromine | 1 | 181.07205 | 2.057 | RP_ ESI+ |
| Theophylline | 1 | 179.05719 | 3.201 | RP_ ESI- |
| Valerylcarnitine | 1 | 246.17021 | 3.055 | RP_ ESI+ |
