# Supplementary material for: Identification of Gut Microbial Lysine and Histidine Degradation and CYP-Dependent Metabolites as Biomarkers of Fatty Liver Disease
Source: mBio. 2023 Jan 30;14(1):e02663-22. doi: 10.1128/mbio.02663-22 (PMC9973343; doi:10.1128/mbio.02663-22)
Supplement: TABLE S3 [file mbio.02663-22-s0009.docx]

**Table S3.** Fecal metabolites and liver fat.

**A)** The fecal metabolites that differed between the low (*n*=14) and high (*n*=25) liver fat groups, and their fold changes between the groups.

| **Metabolite name** | **Fold change high/low liver fat** | **Rank-Sum p** | **p adjusted for metformin use + weight** |
| --- | --- | --- | --- |
| **Endogenous metabolites** |  |  |  |
| 6-beta-Hydroxytestosterone | 0.157 | 0.008 | **0.047** |
| Cholic acid | 3.753 | 0.024 | 0.13 |
| Dimethyluric acid | 0.381 | 0.05 | 0.254 |
| Epinephrine | 0.241 | 0.006 | **0.017** |
| N6,N6,N6-Trimethyllysine | 2.007 | 0.05 | 0.124 |
| N-omega-Acetylhistamine | 7.346 | 0.007 | **0.039** |
| Saccharopine | 2.763 | 0.038 | **0.019** |
| Suberic acid | 0.465 | 0.01 | 0.068 |
| Testosterone sulfate | 20.057 | 0.014 | 0.06 |
| **Metabolites of nutrients** |  |  |  |
| 1,4-Dideoxy-1,4-imino-D-ribitol | 0.392 | 0.043 | 0.108 |
| 2-Ethyl-2-hydroxybutyric acid | 0.339 | 0.046 | 0.085 |
| 2-Hydroxyphenylalanine | 0.356 | 0.019 | **0.045** |
| Hydroxyhexadecanoic acid | 2.097 | 0.05 | 0.096 |
| Ile-Glu | 2.007 | 0.012 | **0.02** |
| Isoleucine | 0.273 | 0.012 | 0.078 |
| Jasmonic acid | 0.473 | 0.04 | 0.107 |
| **Microbial metabolites** |  |  |  |
| Azelaic acid | 0.432 | 0.007 | 0.091 |
| Dodecanedioic acid | 0.309 | 0.026 | **0.034** |
| Hypoxanthine | 0.498 | 0.038 | 0.296 |

* Quade's (non-parametric) Ancova with the use of metformin and body mass (kilograms) as the covariates

**B)** Associations between the fecal metabolites and liver fat percentage. The first two columns show the Spearman correlation coefficient and p-value without adjusting, respectively. The last two columns show the Spearman correlation coefficient and p-value after adjusting for the use of metformin and body weight.

| **Metabolite name** | **Spearman R liver fat** | **p-value liver fat** | **R adjusted*** | **p-value adjusted*** |
| --- | --- | --- | --- | --- |
| **Endogenous metabolites** |  |  |  |  |
| 6beta-Hydroxytestosterone | -0.424 | 0.007 | 0.18 | 0.286 |
| 7alpha,27-Dihydroxycholesterol | 0.401 | 0.011 | -0.288 | 0.084 |
| Anserine | 0.324 | 0.044 | 0.304 | 0.067 |
| Cholic acid | 0.417 | 0.008 | 0.299 | 0.073 |
| Creatinine | 0.357 | 0.026 | 0.204 | 0.227 |
| Epinephrine | -0.338 | 0.035 | -0.269 | 0.107 |
| Histamine | 0.325 | 0.044 | 0.154 | 0.363 |
| Methylimidazoleacetic acid | 0.318 | 0.049 | 0.226 | 0.178 |
| N6,N6,N6-Trimethyllysine | 0.383 | 0.016 | 0.327 | **0.048** |
| N-Acetyl-glutamic acid | -0.322 | 0.045 | 0.272 | 0.103 |
| N-Acetylleucine | 0.362 | 0.024 | 0.37 | **0.024** |
| N-Acetyl-mannosamine | 0.338 | 0.035 | -0.191 | 0.257 |
| N-Acetyl-tyrosine | -0.325 | 0.043 | -0.273 | 0.102 |
| N-omega-Acetylhistamine | 0.42 | 0.008 | 0.166 | 0.325 |
| Suberic acid | -0.42 | 0.008 | -0.277 | 0.097 |
| Trans-Ferulic acid | 0.395 | 0.013 | 0.314 | 0.058 |
| **Exogenous compounds** |  |  |  |  |
| Glycyrrhetinic acid | 0.358 | 0.025 | -0.178 | 0.291 |
| 1-Naphthylamine | 0.36 | 0.024 | 0.295 | 0.076 |
| 3-Amino-4-hydroxybenzoic acid | 0.341 | 0.034 | 0.326 | **0.049** |
| **Microbial metabolites** |  |  |  |  |
| Azelaic acid | -0.396 | 0.013 | 0.266 | 0.112 |
| Dodecanedioic acid | -0.415 | 0.009 | -0.22 | 0.19 |
| Indolelactic acid | 0.342 | 0.033 | 0.331 | 0.045 |
| **Metabolites of Nutrients** |  |  |  |  |
| 1,4-Dideoxy-1,4-imino-D-ribitol | -0.347 | 0.03 | -0.288 | 0.084 |
| 1-Amino-propan-2-ol | 0.371 | 0.02 | 0.552 | **<0.001** |
| 1-Methylhistidine | 0.393 | 0.013 | 0.314 | 0.058 |
| Ala-Pro | 0.317 | 0.05 | 0.268 | 0.109 |
| Galacturonic acid | 0.356 | 0.026 | 0.305 | 0.067 |
| Ile-Glu | 0.365 | 0.022 | 0.34 | **0.04** |
| Jasmonic acid | -0.345 | 0.032 | -0.274 | 0.101 |
| Linoleoyl ethanolamide | 0.359 | 0.025 | 0.194 | 0.25 |
| Oleoylethanolamide | 0.328 | 0.041 | 0.288 | 0.084 |
| p-Hydroxyphenyllactate | 0.436 | 0.005 | 0.354 | **0.032** |
| Pyrrolidine | 0.342 | 0.033 | 0.301 | 0.071 |
| Taurine | 0.382 | 0.016 | 0.299 | 0.072 |

* Partial Spearman correlation with the use of metformin and body mass (kilograms) as the covariates
