# Supplementary material for: Identification of Gut Microbial Lysine and Histidine Degradation and CYP-Dependent Metabolites as Biomarkers of Fatty Liver Disease
Source: mBio. 2023 Jan 30;14(1):e02663-22. doi: 10.1128/mbio.02663-22 (PMC9973343; doi:10.1128/mbio.02663-22)
Supplement: FIG S4 [file mbio.02663-22-s0005.docx]

**S4A
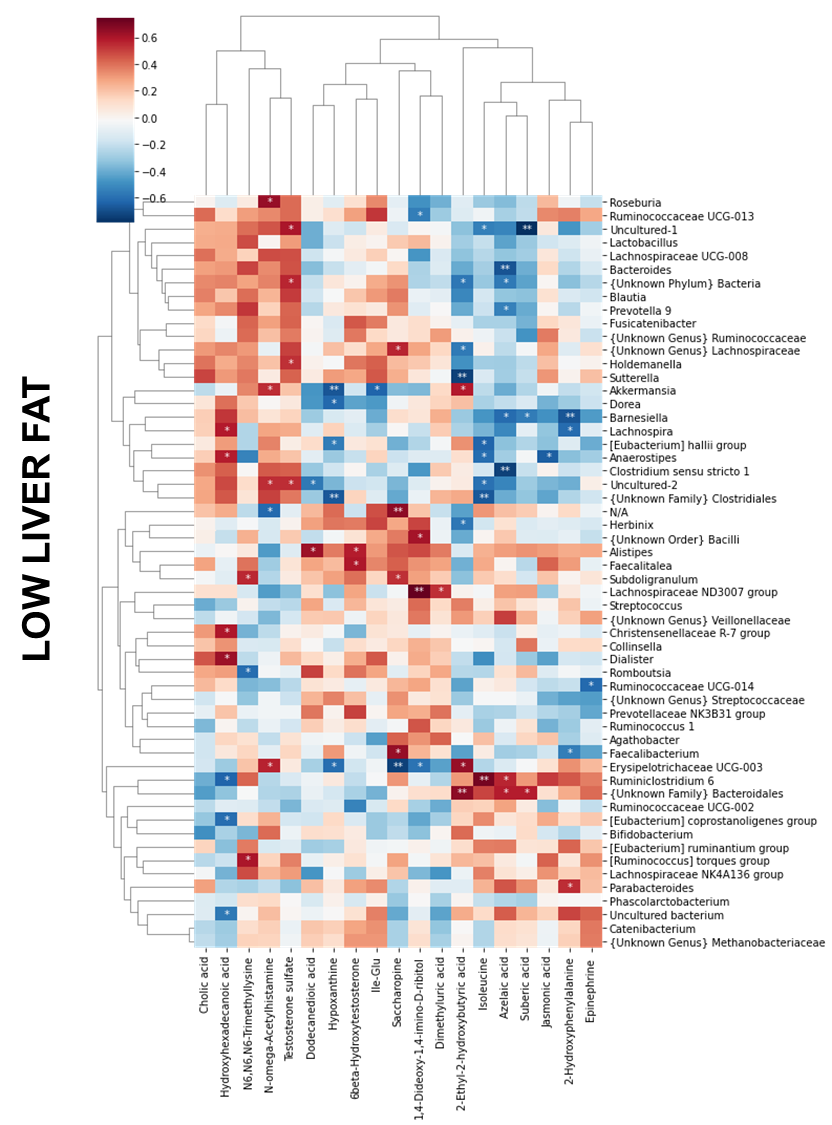
**

**S4B**

**
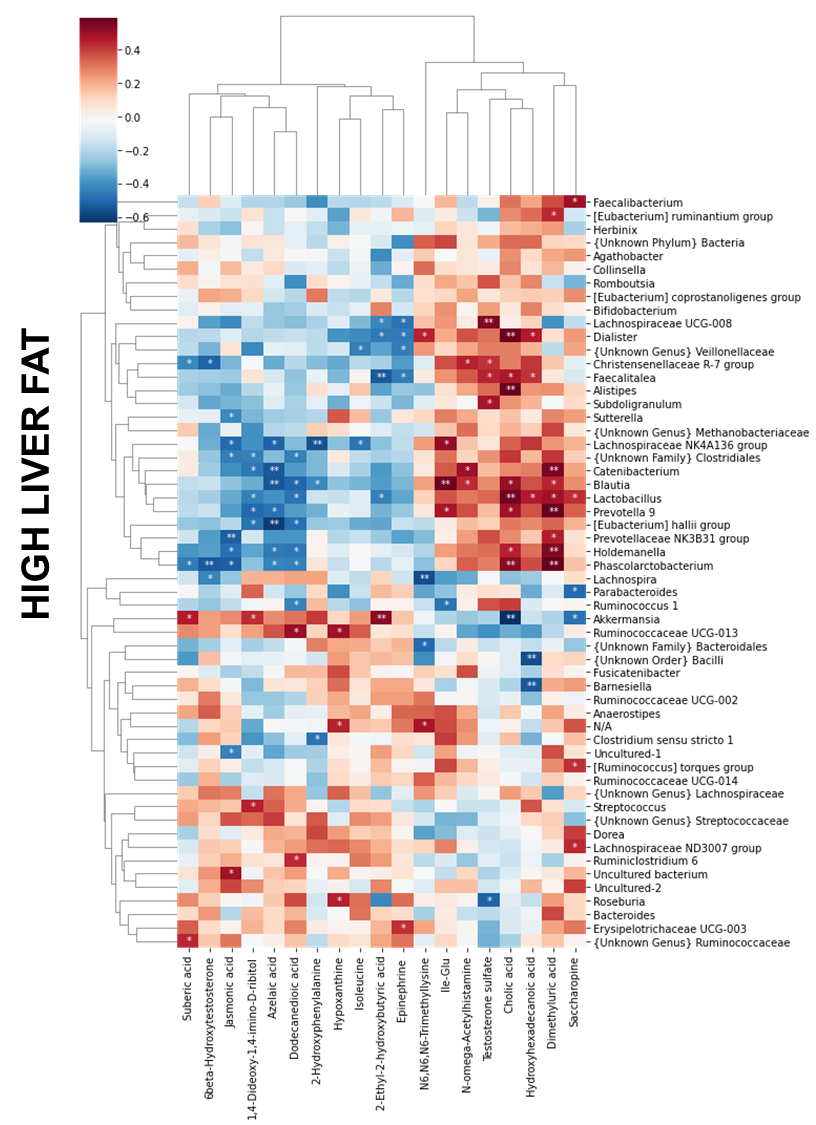
**

**Figure S4.** Visualization of the associations between the gut microbiota genera and fecal metabolites with heatmaps in **(A)** low liver fat group (*n*=25), and (**B)** high liver fat group (*n*=37). Spearman correlation significance * < 0.05 ** < 0.01
