# Supplementary material for: Identification of Gut Microbial Lysine and Histidine Degradation and CYP-Dependent Metabolites as Biomarkers of Fatty Liver Disease
Source: mBio. 2023 Jan 30;14(1):e02663-22. doi: 10.1128/mbio.02663-22 (PMC9973343; doi:10.1128/mbio.02663-22)
Supplement: TABLE S4 [file mbio.02663-22-s0010.docx]

**Table S4.** Plasma metabolites and liver fat.

**A)** The plasma metabolites that differed between low (*n*=11) and high (*n*=7) liver fat groups, and their fold changes between the groups. The second column shows the p-value without adjusting, and the third the p-value after adjusting to body weight. In these study groups there were no participants using metformin, and thus, the use of metformin was not used as covariate as it was in the fecal metabolite analyses.

| **Metabolite** | **Fold change high/low liver fat** | **Rank-sum p-value** | **p-value adjusted for body weight*** |
| --- | --- | --- | --- |
| **Endogenous metabolites** |  |  |  |
| Cholic acid | 2.907 | 0.033 | **0.036** |
| Chenodeoxycholic acid | 2.327 | 0.003 | **0.006** |
| Glycochenodeoxycholic acid | 2.527 | 0.016 | **0.003** |
| Glycocholic acid | 3.855 | 0.016 | **0.001** |
| Glycodeoxycholic acid | 4.301 | 0.013 | **0.003** |
| Paraxanthine | 2.020 | 0.013 | **0.018** |
| Theobromine | 2.004 | 0.026 | 0.055 |
| Theophylline | 2.094 | 0.016 | **0.031** |
| Deoxycholic acid | 3.617 | 0.001 | **0.001** |
| **Exogenous compounds** |  |  |  |
| Cyclo(leucylprolyl) | 3.343 | 0.001 | **0.001** |
| Pyrocatechol sulfate | 0.358 | 0.008 | **0.047** |
| **Microbial metabolites** |  |  |  |
| Indole-3-methyl acetate | 0.460 | 0.042 | 0.120 |
| **Nutrients/ Food constituents** |  |  |  |
| Caffeine | 4.145 | 0.008 | **0.011** |
| Piperine | 2.650 | 0.026 | **0.046** |

* Quade's (non-parametric) Ancova using body mass (kilograms) as the covariate.

**B)** Associations between the plasma metabolites and liver fat percentage. The first two columns show the Spearman correlation coefficient and p-value without adjusting, respectively. The last two columns show the Spearman correlation coefficient and p-value after adjusting for the body weight. In this study group there were no participants using metformin, and, thus the use of metformin was not used as covariate as it was in the fecal metabolite analyses.

| **Metabolite** | **Spearman R liver fat** | **p-value liver fat** | **R adjusted*** | **p-value adjusted*** |
| --- | --- | --- | --- | --- |
| **Endogenous metabolites** |  |  |  |  |
| Androstanediol | 0.513 | 0.029 | 0.135 | 0.605 |
| Cortexolone | 0.546 | 0.019 | 0.341 | 0.181 |
| Chenodeoxycholic acid | 0.722 | 0.001 | 0.687 | **0.002** |
| Glycodeoxycholic acid | 0.533 | 0.023 | 0.729 | **0.001** |
| Kynurenine | 0.528 | 0.024 | 0.146 | 0.575 |
| Propionylcarnitine | 0.577 | 0.012 | 0.574 | **0.016** |
| Taurochenodeoxycholic acid | 0.605 | 0.008 | 0.464 | 0.061 |
| Deoxycholic acid | 0.847 | 0.000 | 0.856 | **0.000** |
| Valerylcarnitine | 0.707 | 0.001 | 0.423 | 0.091 |
| **Exogenous compounds** |  |  |  |  |
| Glycyrrhetinic acid | -0.490 | 0.039 | -0.205 | 0.429 |
| Cyclo(leucylprolyl) | 0.676 | 0.002 | 0.600 | **0.011** |
| Pyrocatechol sulfate | -0.640 | 0.004 | -0.402 | 0.109 |
| **Nutrients** |  |  |  |  |
| Glutamic acid | 0.637 | 0.004 | 0.264 | 0.306 |
| Isoleucine | 0.487 | 0.041 | 0.144 | 0.582 |
| Proline | 0.507 | 0.032 | 0.344 | 0.176 |
| **Phospholipids** |  |  |  |  |
| LysoPC(15:1) | -0.529 | 0.024 | -0.349 | 0.170 |
| LysoPC(17:0) | -0.630 | 0.005 | -0.414 | 0.098 |
| LysoPC(18:0) | -0.563 | 0.015 | -0.312 | 0.224 |
| LysoPC(18:2) | -0.481 | 0.043 | -0.181 | 0.488 |
| LysoPC(22:6) | -0.498 | 0.035 | -0.078 | 0.767 |
| LysoPC(O-18:1) | -0.506 | 0.032 | -0.301 | 0.240 |
| LysoPC(P-16:0) | -0.543 | 0.020 | -0.323 | 0.205 |
| LysoPE(22:6) | -0.481 | 0.043 | -0.134 | 0.607 |
| PC(18:2/17:0) | -0.649 | 0.004 | -0.336 | 0.188 |
| PC(18:2/20:4) | -0.672 | 0.002 | -0.057 | 0.828 |
| PC(20:5/18:2) | -0.665 | 0.003 | -0.284 | 0.268 |
| PC(P-16:0/18:1) | -0.530 | 0.024 | -0.337 | 0.186 |
| PE(18:1e_22:6) | -0.476 | 0.046 | -0.117 | 0.655 |
| SM(d18:1/12:0) | -0.475 | 0.046 | -0.314 | 0.220 |
|  |  |  |  |  |

* Partial Spearman correlation using body mass (kilograms) as the covariate. In this study group there were no participants using metformin. Thus, the use of metformin was not used as covariate as it was for the fecal metabolites.
