# Supplementary material for: Identification of Gut Microbial Lysine and Histidine Degradation and CYP-Dependent Metabolites as Biomarkers of Fatty Liver Disease
Source: mBio. 2023 Jan 30;14(1):e02663-22. doi: 10.1128/mbio.02663-22 (PMC9973343; doi:10.1128/mbio.02663-22)
Supplement: FIG S5 [file mbio.02663-22-s0006.docx]

**S5**

**
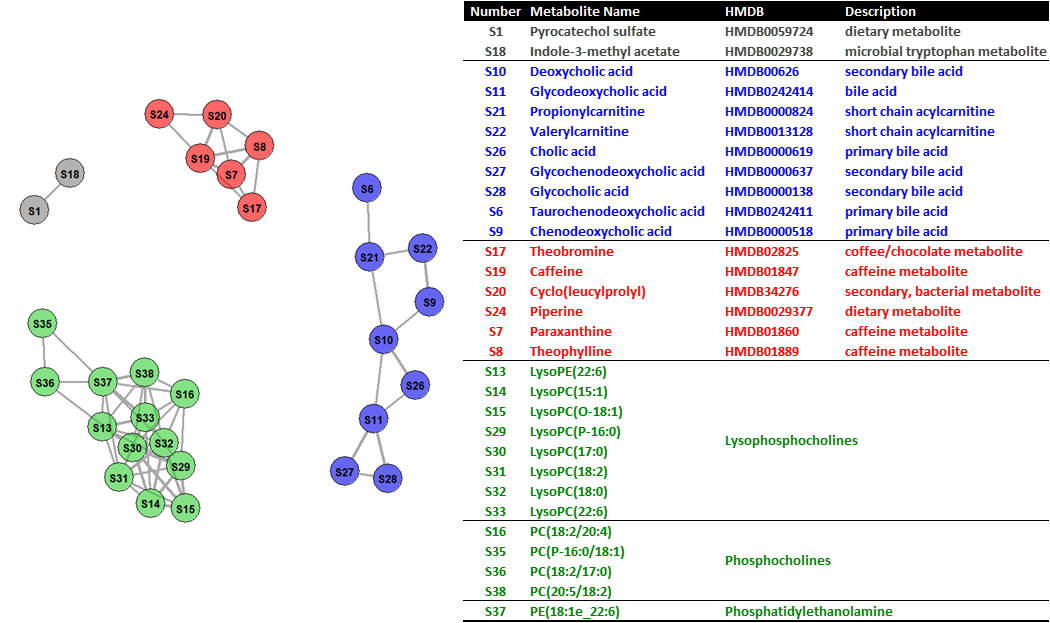
**

**Figure S5. Correlation network community analysis of the serum metabolites.** On the left, a Pearson correlation matrix was computed for all significantly altered serum metabolites. Metabolite correlations with a Pearson coefficient >0.6 and a p-value <0.05 were then subject to the Girvan-Newman algorithm to identify highly connected subgraphs. This analysis revealed four network “communities” represented by the four colors. On the right, the metabolites in each of the four groups along with the associated Human Metabolome Database number and description.
