# Supplementary material for: Identification of Gut Microbial Lysine and Histidine Degradation and CYP-Dependent Metabolites as Biomarkers of Fatty Liver Disease
Source: mBio. 2023 Jan 30;14(1):e02663-22. doi: 10.1128/mbio.02663-22 (PMC9973343; doi:10.1128/mbio.02663-22)
Supplement: TEXT S1 [file mbio.02663-22-s0001.docx]

**SUPPLEMENTARY METHODS**

*Magnetic resonance imaging*

The imaging was performed using Siemens Magnetom Symphony, A TIM system, 1.5T MRI. Measuring the amount of liver fat was performed with the most common technique, out-of-phase/in-phase chemical shift imaging, as described before (<https://doi.org/10.1148/rg.291075123>). A breath–hold technique was applied to avoid breathing-induced artifacts. Two paired gradient echo (GRE) multisectional axial images, which have same repetition time (TR), but different echo time (TE) values were obtained – in-phase (IP) and out-of-phase (OOP) sequences. Liver steatosis is detected as a signal loss in out-of-phase images. For a higher accuracy, out-of-phase images are acquired before the in-phase imaging.

The image data were transferred to a personal computer in Digital Imaging and Communication in Medicine (DICOM) format. Data analysis was carried out using the open-source Osirix DICOM Viewer software. For each subject three elliptical regions of interest were manually drawn at identical liver sites on IP and OOP images, and signal intensity was measured (S_IP_ and S_OP_). For each region of interest, we could calculate the fat signal fraction using the equation: FSF = (S_IP_ - S_OP_) / 2S_IP_. For further data analyses we used an averaged fat signal fraction.

*Magnetic resonance spectroscopy*

Magnetic resonance spectroscopy (MRS) experiments were performed on a 1.5 Tesla clinical imager (AvantoFit, Siemens, Erlangen, Germany).  To determine liver fat content, a 25x25x25 mm^3^ voxel was placed in the middle of the right liver lobe avoiding vascular structures, bile ducts and gall bladder. Point resolved spectroscopy (PRESS) sequence was used for volume selection with TE of 30 msec and 4 averages.  Signal acquisition was triggered to end exhalation using navigator belt to eliminate motion artifacts due to respiratory motion so that TR was kept > 4000 msec.

Liver spectra were analyzed with jMRUI 6.0 software (<https://doi.org/10.1088/0957-0233/20/10/104035>) and intensities of methylene and water resonances were determined using the AMARES algorithm ([https://doi.org/10.1006/jmre.1997.1244](https://doi.org/10.1006/jmre.1997.1244)). Signal intensities were corrected for T2 relaxation effects and liver fat determined as an intensity ratio of methylene/ (methylene + water). Ratios were further converted to mass fractions as described previously (https://doi.org/[10.1053/j.gastro.2009.06.005](https://doi.org/10.1053/j.gastro.2009.06.005) ).

*Gut microbiota 16S rRNA gene sequencing*

The 16S rRNA gene was amplified using primers S-D-Bact-0341-b-S-17, 5´-CCTACGGGNGGCWGCAG-3’ and S-D-Bact-0785-a-A-21, 5´-GACTACHVGGGTATCTAATCC-3´ targeting the V3-V4 regions of the SSU rRNA gene. The PCR reaction contained 1xMaxima™ SYBR Green qPCR Master Mix (Thermo Fisher Scientific, Waltham, MA, USA), 10 µM of primers and 20-80 ng of DNA template. The thermal cycling consisted of 10 min initial denaturation at 95°C, 30 cycles at 94°C 30 sec, +52°C 60 s and 72°C 60 s and final extension at 72°C for 5 min (C1000 ThermalCycler, Bio-Rad Laboratories, Hercules, CA, USA). To add Ion Torrent PGM sequencing adapters and barcodes to the ends of the PCR product, one µl of the PCR product was used as template in the second PCR, where 10 cycles were performed using linker and fusion primers (10 µM of M13_ S-D-Bact-0341-b-S-17, 1 µM of IonA_IonXpressBarcode_M13 and P1_ S-D-Bact-0785-a-A-21), with conditions otherwise identical to the first amplification. Then, the PCR products were purified with AMPure XP (Beckman Coulter, Brea, USA), quantified with PicoGreen (Quant-iT™ PicoGreen™ dsDNA Assay Kit, Thermo Fisher Scientific, Waltham, USA), and pooled in equimolar quantities for sequencing on Ion Torrent PGM (Thermo Fisher Scientific) using Hi-Q View OT2 Kit for emulsion PCR, Hi-Q View Sequencing Kit for the sequencing reaction, and Ion 318 Chip v2 (Thermo Fisher Scientific).

*Plasma and fecal metabolomic analyses* *from subsets of study participants*

A pooled sample from biological samples per experiment was injected at the beginning of the sequence to equilibrate the analytical platform and then after every 12 samples throughout the analysis for quality control (QC). In addition, a solvent blank was prepared and injected at the beginning of the sequence. Samples were randomized before the analysis.

Non-targeted metabolite profiling analysis of feces (low liver fat (LF) *n*=14, high liver fat (HF) *n*=25) and plasma (low liver fat (LF) *n*=11, high liver fat (HF) *n*=7) was carried out at the LC-MS metabolomics center (Biocenter Kuopio, University of Eastern Finland). The analysis was carried out using an ultra-high performance liquid chromatography (Vanquich Flex UHPLC system, Thermo Scientific, Bremen, Germany) coupled online to a high-resolution mass spectrometry (Q Exactive Focus, Thermo Scientific, Bremen, Germany). Samples were analyzed using a reversed phase (RP) and a hydrophilic interaction chromatography (HILIC) technique. The sample solutions (2 µl for serum, 1 µl for feces) was injected onto a RP column (Zorbax Eclipse XDBC18, 2.1 × 100mm, 1.8 μm, Agilent Technologies, Palo Alto, CA, USA) that was kept at 40 °C. In HILIC, the sample solution (2 µl) was injected onto a column (Acquity UPLC® BEH Amide 1.7 µm, 2.1x100 mm, Waters Corporation, Milford, MA, USA) that was kept at 45 °C. In RP technique the mobile phases, delivered at 400 μL/min, consisted of water (eluent A) and methanol (eluent B), both containing 0.1 % (v/v) of formic acid. The following gradient profile was used: 0–10 min: 2 to 100% B, 10–14.50 min: 100% B, 14.50–14.51 min: 100 to 2% B; 14.51–20 min: 2% B. In HILIC, the mobile phases, delivered at 600 μL/min, consisted of 50% (v/v) (eluent A) and 90% (v/v) (eluent B) acetonitrile, respectively, both containing 20 mM ammonium formate (pH 3). Following gradient profile was used: 100% B (0-2.5 min); 100%→0% B (2.5-10 min); 0%→100% B (10-10.01 min); 100% B (10.01-12.5 min). The sample tray was at 10 °C during these analyses.

A mass spectrometry was equipped with a heated electrospray ionization and the positive ionization mode was used to acquire the data. The following ESI source settings were utilized; spray voltage 3.5 kV for positive and 3.0 kV for negative ionization modes, sheath gas (40), auxiliary gas (10), and sweep gas (2) (flow rates as arbitrary units for ion source). The capillary temperature and the probe heater temperature were both set to 300 °C. The S-lens RF level was set to 50 V. A full scan range from 120 to 1100 (*m/z*) and from 70 to 750 (m/z) were used with the resolution of 70,000 (m/Δm, full width at half maximum at 200 u) in RP and HILIC techniques, respectively. The automatic injection time was used, and Automated Gain Control (AGC) was targeted at 1,000,000 ions. The detector was calibrated before the sample sequence and subsequently operated at high mass accuracy (<2 ppm).

For the data-dependent product ion spectrum (MS2) experiments, the Q-Exactive spectrometer was used with the same source parameters and chromatography conditions as previously described. Two scan events were used: (i) an MS scan with a mass resolution power, AGC target and maximum injection time set to 35,000 (m/Δm, full width at half maximum at 200 u), 1,000,000 ions and automatic, respectively, and (ii) an MS2 scan (in HCD mode) at a normalized collision energy ranging from 20 to 40 %, depending on the molecule, with a mass resolution power, AGC target, maximum injection time, isolation window set to 17,500 (m/Δm, full width at half maximum at 200 u), 50,000, automatic and 1.5 *m/z*, respectively. Loop count was 3, apex trigger 0.2 to 3 s, and dynamic exclusion 15 sec.

The HILIC analysis of plasma was performed using an ultra-high-performance liquid chromatography quadrupole-time-of-flight mass spectrometry (UHPLC-qTOF-MS) system (Agilent Technologies, Waldbronn, Karlsruhe, Germany), which consisted of a 1290 LC system, a Jetstream electrospray ionization (ESI) source, and a 6540 ultra-high-definition accurate-mass qTOF-MS. The data was acquired in both ionization polarities: ESI positive (ESI+) and ESI negative (ESI–). The temperature of the sample tray was maintained at +10°C. The data acquisition software was the MassHunter Acquisition B.04.00 (Agilent Technologies).

In the HILIC method, 2 μl of the sample solution was injected onto a column (Acquity UPLC BEH Amide column, 2.1 × 100 mm, 1.7 μm, Waters Corporation, Milford, MA, USA) kept at +45°C. Mobile phases, delivered at 600 µL/min, consisted of 50% (v/v, eluent A) and 90% (v/v, eluent B) ACN, both containing 20 mM NH_4_HCO_2_ (pH 3). The following gradient profile was used: 0–2.5 min: 100% eluent B, 2.5–10 min: 100 → 0% eluent B, 10–10.01 min: 0 → 100% eluent B; 10.01–12.5 min: 100% eluent B.

A Jet Stream ESI source, operated in both positive and negative ionization modes, was used under the following conditions: drying gas temperature +325°C and a flow of 10 L/min, sheath gas temperature +350°C and a flow of 11 L/min, nebulizer pressure 45 psi, capillary voltage 3500 V, nozzle voltage 1000 V, fragmentor voltage 100 V, and skimmer 45 V. N_2_ was used as the instrument gas. For data acquisition, a 2 GHz extended dynamic range mode was utilized in both positive and negative ion modes from *m/z* 50 to 1600. The data were collected in the centroid mode at an acquisition rate of 1.7 spectra/s (599 ms/spectrum) with an abundance threshold of 150. For the automatic data-dependent MS/MS analyses, the precursor isolation width was 1.3 Da, and from every precursor a scan cycle of 4 most abundant ions was selected for fragmentation. These ions were excluded after 2 product ion spectra and released again for fragmentation after a 0.25 min hold. Precursor scan time was based on ion intensity, ending at 25,000 counts or after 300 ms. The product ion scan time was 300 ms, and the collision energies were 10, 20, and 40 V in subsequent runs. The TOF was calibrated on a daily basis and operated at high accuracy (<2 ppm). Continuous mass axis calibration was performed by monitoring 2 reference ions from an infusion solution throughout the runs. The reference ions were *m/z* 121.050873 and *m/z* 922.009798 in the positive mode and *m/z* 112.985587 and *m/z* 966.000725 in the negative mode, respectively.

For the fecal and seral MS-data, peak picking and alignment were done using MS-Dial. Aligned raw peak areas were exported as spreadsheets. Further, quality control, drift correction and feature clustering were done using R, as described previously. (<https://doi.org/10.3390/metabo10040135>). The raw abundances (areas) of the molecular features were log2-transformed and the group differences were analyzed using a nonparametric test (Mann-Whitney U). Features with a p < 0.05 and a fold change of at least 2 between the groups were considered of interest and further clustered into metabolites based on physicochemical similarity. Annotations of the metabolites of interest were generated based on accurate mass and isotope information; i.e., ratios, abundances, and spacing, as well as product ion spectra (MS2) against existing libraries, either in-house for level I or online spectral databases for levels II-III according to the guidelines from the Metabolomics Standard Initiative (<https://doi.org/10.1007/s11306-007-0082-2>).
